# The impact of androgen-induced translation in modulating androgen receptor activity

**DOI:** 10.1186/s13062-024-00550-6

**Published:** 2024-11-11

**Authors:** Justus S. Israel, Laura-Maria Marcelin, Sherif Mehralivand, Jana Scholze, Jörg Hofmann, Matthias B. Stope, Martin Puhr, Christian Thomas, Holger H. H. Erb

**Affiliations:** 1 Department of Urology, Faculty of Medicine, University Hospital Carl Gustav Carus, Technische Universität Dresden, 01307 Dresden, Germany; 2 German Society of Urology, UroFors Consortium (Natural Scientists in Urological Research), 14163 Berlin, Germany; 3 Department of Gynecology and Gynecological Oncology, University Hospital Bonn, 53127 Bonn, Germany; 4 Department of Urology, Medical University of Innsbruck, 6020 Innsbruck, Austria; 5 German Cancer Consortium (DKTK), Partner Site Dresden and German Cancer Research Center (DKFZ), 69120 Heidelberg, Germany; 6 Universitätsklinikum Carl Gustav Carus an der Technischen Universität Dresden, Technischen Universität Dresden, Fetscherstraße 74, 01307 Dresden, Germany

**Keywords:** AR, PCa, NR3C4, Androgen deprivation therapy

## Abstract

**Introduction:**

Dysregulated androgen receptor (AR) activity is central to various diseases, particularly prostate cancer (PCa), in which it drives tumour initiation and progression. Consequently, antagonising AR activity via anti-androgens is an indispensable treatment option for metastatic PCa. However, despite initial tumour remission, drug resistance occurs. Therefore, the AR signalling pathway has been intensively investigated. However, the role of AR protein stability in AR signalling and therapy resistance has not yet been deciphered. Therefore, this study aimed to investigate the role of AR protein changes in transactivity and assess its mechanism as a possible target in PCa.

**Methods:**

LNCaP, C4-2, and 22Rv1 cells were treated with R1881, enzalutamide, cycloheximide, and Rocaglamide. Mass spectrometry analyses were performed on LNCaP cells to identify the pathways enriched by the treatments. Western blotting was performed to investigate AR protein levels and localisation changes. Changes in AR transactivity were determined by qPCR.

**Results:**

Mass spectrometry analyses were performed on LNCaP cells to decipher the molecular mechanisms underlying androgen- and antiandrogen-induced alterations in the AR protein. Pathway analysis revealed the enrichment of proteins involved in different pathways that regulate translation. Translational and proteasome inhibitor experiments revealed that these AR protein changes were attributable to modifications in translational activity. Interestingly, the effects on AR protein levels in castration-resistant PCa (CRPC) cells C4-2 or enzalutamide-resistant cells 22Rv1 were less prominent and non-existent. This outcome was similarly observed in the alteration of AR transactivation, which was suppressed in hormone-sensitive prostate cancer (HSPC) LNCaP cells by translational inhibition, akin to the effect of enzalutamide. In contrast, treatment-resistant cell lines showed only a slight change in AR transcription.

**Conclusion:**

This study suggests that in HSPC, AR activation triggers a signalling cascade that increases AR protein levels by enhancing its translation rate, thereby amplifying AR activity. However, this mechanism appears to be dysregulated in castration-resistant PCa cells.

**Supplementary Information:**

The online version contains supplementary material available at 10.1186/s13062-024-00550-6.

## Background

The androgen receptor (AR), also known as "nuclear receptor superfamily 3 group C number 4", is a ligand-dependent transcription factor belonging to the nuclear receptor protein superfamily [1, 2]. The AR has a molecular weight of 110–114 kDa, consists of 910–919 amino acids, and is encoded on the X chromosome (Xq11.2–q12) with 8 exons [3]. The AR mediates the biological effects of androgens, including dehydroepiandrosterone, androstenedione, androstenediol, androsterone, testosterone, and dihydrotestosterone. The central role of the AR is in the differentiation of luminal prostate epithelial cells and the regulation of gene expression necessary for prostate function, growth, and survival [4]. Beyond its role in the prostate, the AR is crucial for maintaining muscle, bone, and adipose tissue [5]. In addition to its role in the development of prostate tissue, the AR can also be involved in the pathogenesis of various diseases. These include androgen insensitivity syndrome, spinal and bulbar muscular atrophy, hypogonadism, and benign prostatic hyperplasia (BPH) [6, 7]. Moreover, AR is critical for the development, progression, and treatment of prostate cancer (PCa) [3, 8].

In its inactive state, the AR binds to heat shock protein (HSP)s in the cytoplasm. The binding of androgens induces a conformational change in the AR, leading to hyperphosphorylation and the subsequent release of activated AR from the HSPs [1, 3]. The activated AR then translocates to the nucleus, where the AR-ligand complex dimerises, associates with co-activators, and binds to specific androgen response elements (ARE) on the DNA [1, 3, 9, 10]. This binding to the ARE enables direct interaction with the target gene promoter regions.

Several non-DNA binding-dependent AR actions have been described. These actions are commonly called 'non-genomic,' 'non-classical,' or 'non-canonical' AR signalling [5, 11]. The activation of secondary messenger pathways, including ERK, Akt, and MAPK, has been observed in vitro [5]. Moreover, androgen treatment increased AR protein synthesis in PCa cell lines [12]. These effects occur within seconds to minutes of androgen treatment, indicating that they are too rapid to be attributed to the DNA binding of AR and regulation of the transcription and translation of target genes.

The AR plays a crucial role in PCa, a leading cause of cancer-related deaths in men [13]. Dysregulated AR activity in PCa drives tumour initiation, growth, and progression [14]. Therefore, targeting AR activity through androgen deprivation therapy or anti-androgens (AA, e.g., enzalutamide) has become a vital treatment approach for managing metastatic PCa, offering a promising strategy to address this aggressive and often deadly disease [15]. Despite an initial period of efficacy, anti-androgens are only effective for a limited duration, with the emergence of resistance to therapy and tumour recurrence [14, 16]. Although several molecular adaptations have been identified, the underlying resistance mechanisms remain poorly understood. These include the AR amplification and mutation and the AR protein's increased stability. In vitro data has demonstrated that cells treated with anti-androgens exhibit reduced AR protein levels [17]. The antiandrogen-mediated change in AR protein levels commences as early as two hours after treatment. The extent of the AR protein change correlates with the AR activity and cell viability change. Consequently, AR protein stabilisation contributes to developing resistance to PCa therapy. Therefore, this study aimed to investigate the role of AR protein changes in transactivity and assess their mechanism as a possible target in PCa.

## Materials and methods

### Chemicals

The chemicals listed in Supplementary Table 1 were used at concentrations, as indicated in the results section and figure legends.

### Cell culture

The human PCa cell lines LNCaP and 22Rv1 were obtained from the American Type Culture Collection (ATCC, Manassas, VA, USA). C4-2 cells were kindly provided by Prof. Thalmann (University of Berne, Switzerland) [18]. Dr A. Cato (University of Karlsruhe, Karlsruhe, Germany) provided the cell line LAPC4. LNCaP, C4-2, and LAPC4 cells were cultured as described previously [19]. 22Rv1 cells were cultured in RPMI1640 (Cat# 52,400–025, Thermo Fisher Scientific, Dreieich, Germany) with 10% fetal bovine serum (FBS, Cat# 10,270,106, Thermo Fisher Scientific, Dreieich, Germany). Mycoplasma testing was routinely performed using the Mycoalert Detection Assay (Cat# LT07-318; Lonza, Basel, Switzerland). Cell line authentication was performed yearly using STR profiling.

### Live cell count

For cell counting, the Muse Count & Viability Assay Kit (Cat# MCH600103, Luminex Corp, Austin, TX, USA) was used according to the manufacturer's instructions and analysed using a Muse Cell Analyzer (Luminex Corp).

### Lentiviral transduction

For nuclei counting using the IncuCyte S3 live-cell imaging system, the cell lines were stably transduced with mKATE2-NLS. Lentiviral production was performed as described, and cells were subsequently selected with Blasticidin (10 µg/mL) [20]. The percentage of positive cells was determined using a Compact Fluorescence Microscope BZ-X800E (Keyence, Osaka, Japan) and analysed using BZ-X800 analysis software (Keyence).

### Proliferation assay with the IncuCyte S3 live cell analysis system

Cell proliferation was measured by mKATE2 labelled nuclei counting and confluence determination using the IncuCyte S3 Live-Cell Imaging System (Sartorius AG, Goettingen, Germany). The cells were seeded in 96-well clear flat-bottom plates (Cat# 3596, Corning GmbH, Kaiserslautern, Germany) and incubated overnight at 37 °C with 5% CO_2_. Subsequently, cells were starved overnight in RPMI1640 (Thermo Fisher Scientific) without FBS. Subsequently, the plates were treated and placed into the IncuCyte S3 Live-Cell Imaging System live imaging system (Sartorius AG) and scanned every 6 h. Confluence and cell number were analysed using IncuCyte 2023C analysis software (Sartorius AG) by measuring the growth area or counting the mKATE2 labelled nuclei. Cell proliferation was expressed as increased cell confluence or number compared to the first scan time point or as an x-fold of DMSO-treated controls.

### Western blot

Cell harvesting, protein determination, and western blotting were performed as described earlier [19]. Revert 520 Total Protein Stain Kit (Cat# 926-10016, LI-COR Biosciences, Lincoln, USA) was used for normalisation. Chameleon^®^ Duo Pre-stained Protein Ladder (Cat# 928-60000, LI-COR Biosciences) was used for molecular weight estimation. Signals were acquired using an Odyssey M (LI-COR Biosciences). The uncropped western blot images are shown in the supplementary files. Densitometric analysis was performed using the Empiria Studio 3.2 (LI-COR Biosciences) and normalised to total protein. Supplementary Table 2 lists all the antibodies used, including their company name and the dilutions applied. Uncropped western blot images are displayed in the supplement figures.

### Total RNA isolation, cDNA synthesis, and quantitative real-time PCR (qPCR)

According to the manufacturer's instructions, total RNA was isolated using the DIRECT-ZOL RNA MINIPREP (Cat# R2052, Zymo Research, Freiburg, Germany). Superscript II RNase H Reverse Transcriptase kit (Cat# 18064071, Thermo Fisher Scientific) or iScript™ Reverse Transcription Supermix (Cat# 1708841, Bio-Rad Laboratories GmbH, Feldkirchen, Germany) were used for cDNA synthesis with 500 ng total RNA. qPCR was performed on a CFX Opus 96 Real-Time PCR System (Cat# 12011319, Bio-Rad Laboratories GmbH) using the SsoAdvanced Universal Probes Supermix (Cat# 1725282, Bio-Rad Laboratories GmbH). The CFX Maestro software 2.0 (Bio-Rad Laboratories GmbH) was used to determine cycle threshold (Ct) values by the regression method. Delta (Δ)Ct = Ct_GOI_-Ct_Housekeeper_ values were calculated and expressed as relative mRNA expression (2^−ΔCt^) or relative changes in gene expression (2^−ΔΔCt^) [21, 22]. Following primer assays have been used: *AR* (Cat# 4,351,370, Hs00171172_m1, Thermo Fisher Scientific), *PSA/KLK3* (Cat# 4,351,370, Hs02576345_m1, Thermo Fisher Scientific), *HPRT1* (Cat# 10,031,231, Bio-Rad Laboratories GmbH).

### Data preparation, imputation, overrepresentation analysis (*ORA*), and gene set enrichment analysis (GSEA) of the mass spectrometry data

Cells were lysed using RIPA buffer for Mass Spectrometry, and the protein concentration was determined as described previously [23, 24]. Lysis buffer (10% SDS, 100 mM TEAB, pH 8.5) was added to the samples at a ratio of 1:1. The core facility "Mass Spectrometry and Proteomics TU Dresden" processed and normalised the samples as previously described [25, 26]. Further analyses were performed using R v4.4.1 and R Studio [27, 28]. For further analysis, the gene names were separated and filtered for missing values, and duplicated genes were excluded. The imputation was performed using the missForest package [29]. Imputed data were processed using the limma package, which uses moderated t-statistics and Benjamin–Hochberg multiple analysis correction [30]. To explore the data, Volcano Plots were computed, which visualised significantly downregulated genes with a *p* value of < 0.05, a logFC of < 0, or significantly upregulated genes with a *p* value of < 0.05, and a logFC of > 0. For GSEA, all differentially expressed genes were ranked according to sign(logFC) × log10(adjusted *p* value) and sorted in descending order. Gene symbols were converted to Entrez IDs using clusterProfiler's bitr function [31, 32]. This ranked list was processed by the ReactomePA using the function gsePathway with the parameter by = 'fgsea' [33, 34]. Pathways with an adjusted *p* value < 0.05 were considered significant. Afterwards, a pathway heatmap was created, visualising the NES value of all significant pathways, summarised under their top-level pathway of the Reactome database. Finally, a table representing all significant pathways following/downstream the translation pathway (R-HSA-72766) of the Reactome database areis shown [35–37]. The R script, session information, and used packages were deposited in GitHub at https://doi.org/10.5281/zenodo.13769868.

### Statistics

GraphPad Prism 10.3 (GraphPad Software, San Diego, CA, USA) was used for all statistical analyses, including curve fitting, statistical tests, and plotting. Data are presented as mean ± SEM or mean ± SD to estimate the various means in repeated experiments. Unless otherwise noted, all experiments were performed with at least three biological replicates. Student's t-test (two-sided) and one-way and two-way analyses of variance (ANOVA) were used to identify significant differences. Statistical significance was set at *p* value ≤ 0.05. All differences highlighted by asterisks are statistically significant, as encoded in the figure legends (*p ≤ 0.05; **p ≤ 0.01; ***p ≤ 0.001).

## Results

### Proteasome inhibition does not reverse antiandrogen-induced androgen receptor protein reduction

**Fig. 1:**
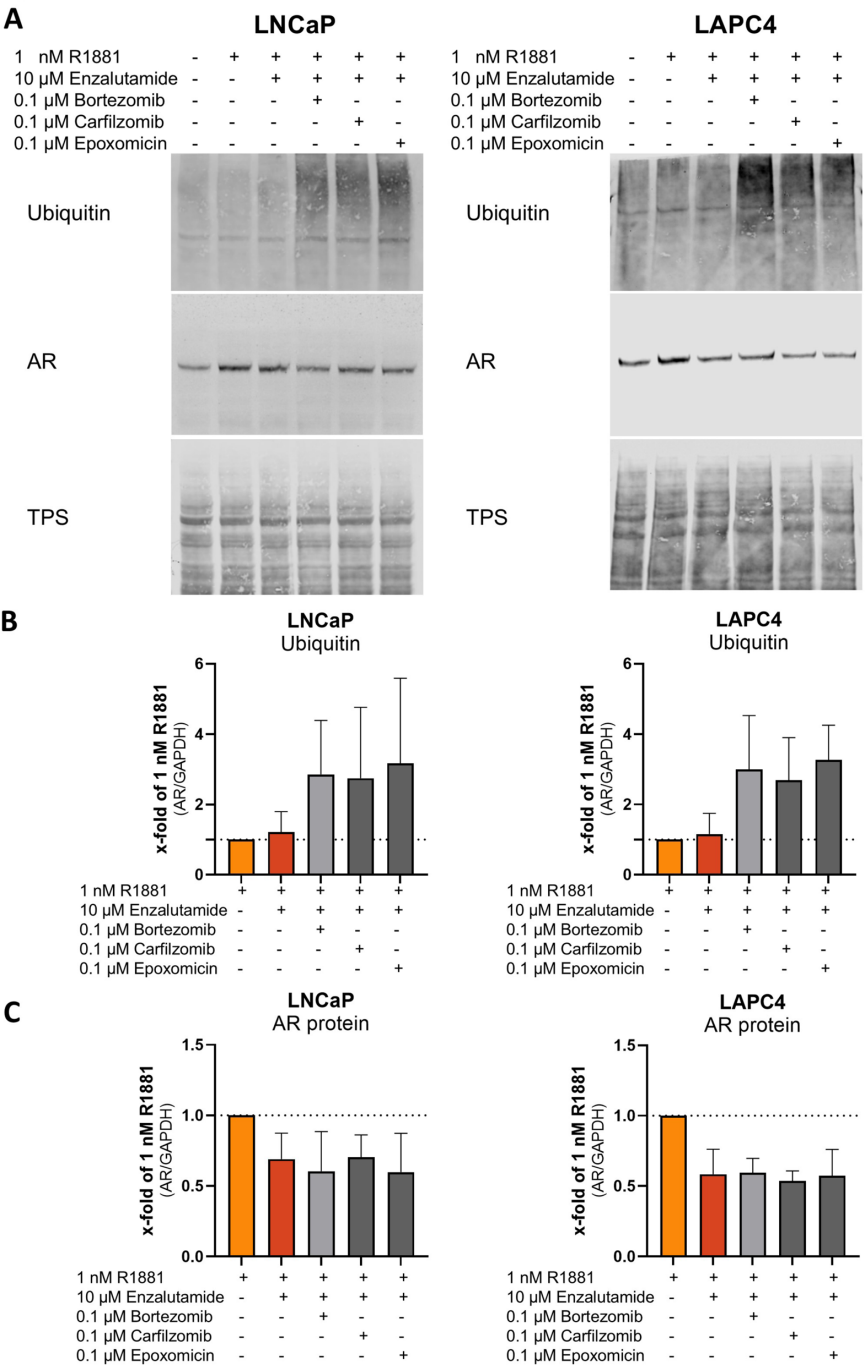
Proteasomal inhibition does not influence enzalutamide-induced AR protein reduction. **A** Representative western blot to investigate the role of proteasomal inhibitors on the AR protein decreasing effect of Enzalutamide, hormone-sensitive PCa cells were treated with 1 nM R1881, 1 nM R1881 + 10 µM Enzalutamide, and 1 µM R1881 + 0.1 µM Bortezomib, Carfilzomib, or Epoxomicin. Ubiqitine was assessed for proteasomal activity. Androgen receptor (AR) levels were normalised to total protein stain (TPS) to assess changes in AR levels after treatment. Uncropped western blots are displayed in Supplementary Fig. 2A. **B** Densitometry of ubiquitin levels relative to TPS. Relative expression levels compared to CTRL after treatment were shown as mean ± SD of four independent experiments. **C** Densitometry of AR protein levels relative to TPS. Relative expression levels compared to CTRL after treatment were shown as mean ± SD of four independent experiments

To investigate whether the proteasome mediates AA-induced AR protein reduction, a working concentration of the proteasome inhibitors bortezomib, carfilzomib, epoxomicin, and (R)-MG-132 was established. Dose–response analysis revealed that the proteasome inhibitors bortezomib, carfilzomib, and epoxomicin effectively reduced cell proliferation (Supplementary Fig. 1A) with an IC_50_ around 0.01 µM (Supplementary Table 3). (R)-MG-132 had minimal effects on LNCaP and LAPC4 cells at the selected concentrations (Supplementary Fig. 1A, Supplementary Table 3). Therefore, they were excluded from this study. After 6 h, dose–response analysis revealed a marginal influence on cell proliferation of up to 0.1 µM (Supplementary Fig. 1B). Western blot analysis revealed that treatment of the cells with 0.1 µM increased ubiquitin in all tested cell lines (Supplementary Fig. 1C). Therefore, 0.1 µM was chosen for rescue experiments. To assess the influence of the proteasome on AA-induced AR protein, 350,000 cells/well were seeded for 24 h, followed by a 24 h starvation step in RPMI1640 without FBS. Subsequently, the cells were treated with DMSO (CTRL), 1 nM R1881, 1 nM R1881 + 10 µM enzalutamide (Enza), or 1 nM R1881 + 10 µM Enza + 0.1 µM proteasome inhibitor for 6 h (Fig. 1). Western blot analysis of the treated cells revealed that proteasome inhibitors induced intracellular ubiquitin levels (Fig. 1A and B) but could not prevent the AA-induced AR protein reduction (Fig. 1A and C). A study by Lin, H. and C. Chang suggested that PTEN promotes AR degradation through the caspase-3-dependent pathway [38]. Therefore, caspase-3 activity in LNCaP cells was assessed for 24 h after treatment with DMSO (CTRL), 1 nM R1881, 1 nM R1881 + 10 µM Enza, and 200 µg/ml cycloheximide (C6) as positive control (Supplementary Fig. 1D). Besides the positive control, none of the treatments could induce caspase 3 activity. These results indicate that the protein degradation is not involved in the AA-induced AR protein reduction in PCa cells.

### R1881 treatment increases protein levels involved in translation.

**Fig. 2:**
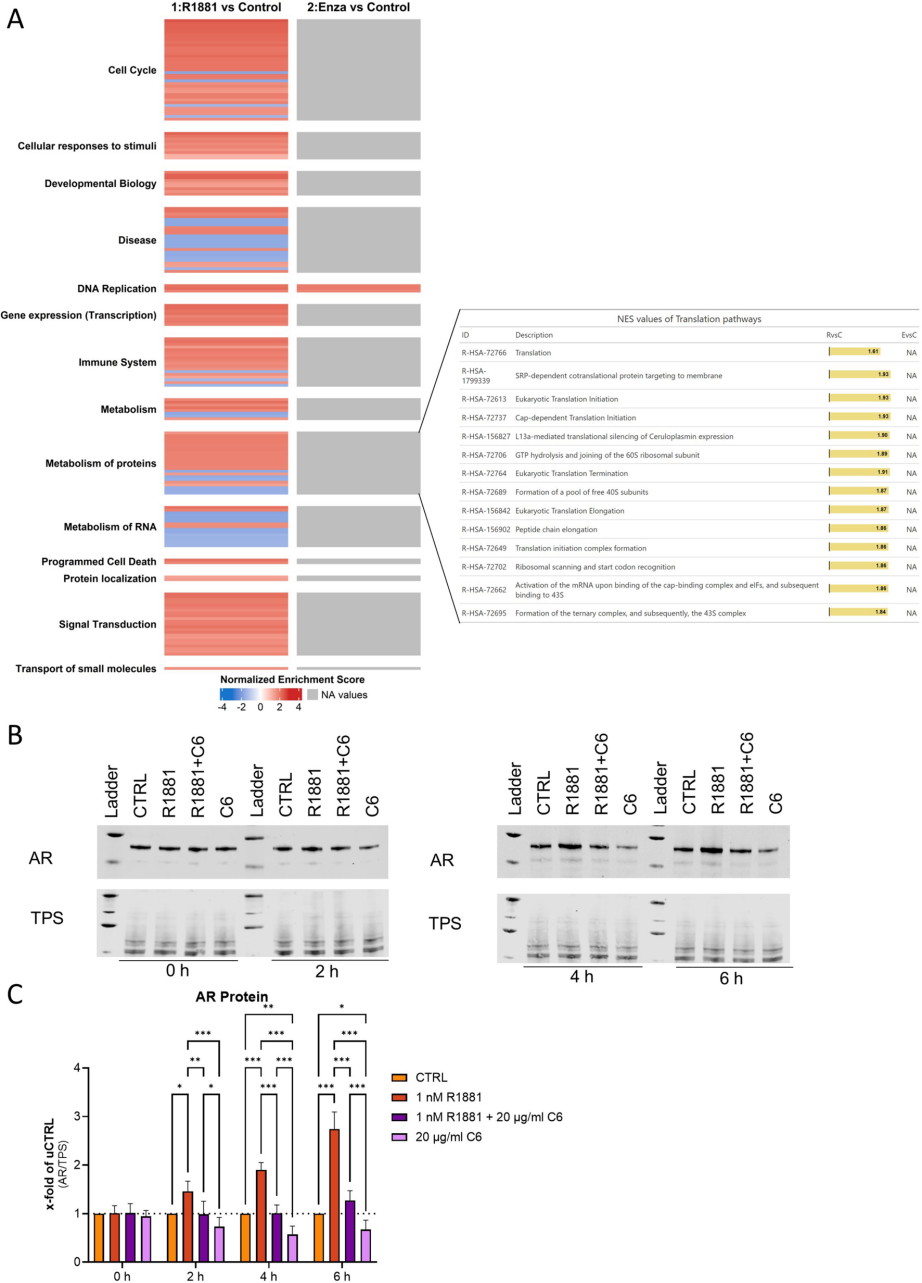
R1881 of LNCaP cells treatment increases protein levels involved in translation. **A** Differentially expressed genes from 1 nM R1881, 1 nM R1881 + 10 µM enzalutamide treatment compared to DMSO were ranked based on their expression value and significance. GSEA was performed using the pre-ranked protein lists, and a heatmap was generated based on the normalised enrichment score and significance of GSEA. Translational pathways were enlarged to show details. **B** Representative western blot of the influence of DMSO (CTRL), 1 nM R1881 treated LNCaP cells combined with DMSO (vehicle control) or 20 µg/ml Cycloheximide (C6), and 20 µg/ml Cycloheximide (C6) alone in LNCaP cells after 0 h, 2 h, 4 h, and 6 h. Uncropped western blots are displayed in Supplementary Fig. 3. **C** Densitometry of AR protein levels relative to TPS. Relative expression levels compared to CTRL after treatment were shown as mean ± SD of five independent experiments. All differences highlighted by asterisks were statistically significant (*: *p* ≤ 0.05; **: *p* ≤ 0.01; ***: *p* ≤ 0.001)

As proteasomal inhibitors could not rescue the AA-induced AR protein reduction in PCa cells, proteomic analysis was performed after treatment with DMSO (CTRL), 1 nM R1881, 1 nM R1881 + 10 µM Enza. To investigate the differential gene expression induced by 1 nM R1881 and 1 nM R1881 + 10 µM Enza after 6 h of treatment, volcano plots for each treatment (Supplementary Fig. 2C and D) in comparison against CTRL were created to illustrate the relationship between fold change and statistical significance (adjusted *p* value) for all analysed genes. Treatment with 1 nM R1881 resulted in a significant change (adjusted *p* value < 0.05) in 354 proteins, whereas Enza reduced this change to 164 proteins. Proteomic analysis also validated Enza's prevention of the R1881-induced AR increase (Supplementary Fig. 2C and D). GSEA, using the Reactome Database after R1881 treatment, revealed the enrichment of multiple gene sets involved in androgen response, including cell cycle, metabolism, RNA and protein metabolism, signal transduction, cellular responses to stimuli, and gene expression (Fig. 2A). This enrichment was inhibited by Enza treatment. As demonstrated, changes in *AR* mRNA did not account for changes in AR protein levels induced by R1881 and Enza. Additionally, as shown here (Fig. 1) the proteasome is not involved in this regulation. Consequently, translation pathway gene sets have also been investigated [12, 17]. The translation pathway gene set exhibited a significantly increased normalised enrichment score (NES) for all pathways. These results indicated a significant overrepresentation of proteins involved in translational control among the upregulated R1881-treated LNCaP cells (Fig. 2C). None of these pathways were significantly enriched when treated with Enza. These results highlight the potential role of translation in AR regulation.

To validate the involvement of the changes in translational pathways in the R1881-induced increase in AR protein levels, cells were treated for 0 h, 2 h, 4 h, and 6 h with DMSO, 1 nM R1881, 1 nM R1881 + 20 µg/ml cycloheximide (C6), and 20 µg/ml C6 alone, and changes in AR protein levels were assessed by western blot analysis (Fig. 2 E and F). To ensure that C6 did not significantly alter the total protein levels, LNCaP cells were treated with DMSO or 20 µg/ml C6 for 6 h, and the protein concentration per cell and total protein stain/cell after western blotting were compared (Supplementary Fig. 2E and F), showing no differences between the treatments. The time series experiment revealed that at 2 h R1881 significantly increased the AR protein levels, whereas C6 prevented the R1881-induced increase in AR protein levels (Fig. 2 F). Moreover, C6 treatment alone significantly decreased the AR protein levels after 4 h (Fig. 2 F), resulting in a half-life of 2.1 h.

### Inhibition of translation with cycloheximide prevents increased androgen receptor protein levels and transactivity

**Fig. 3:**
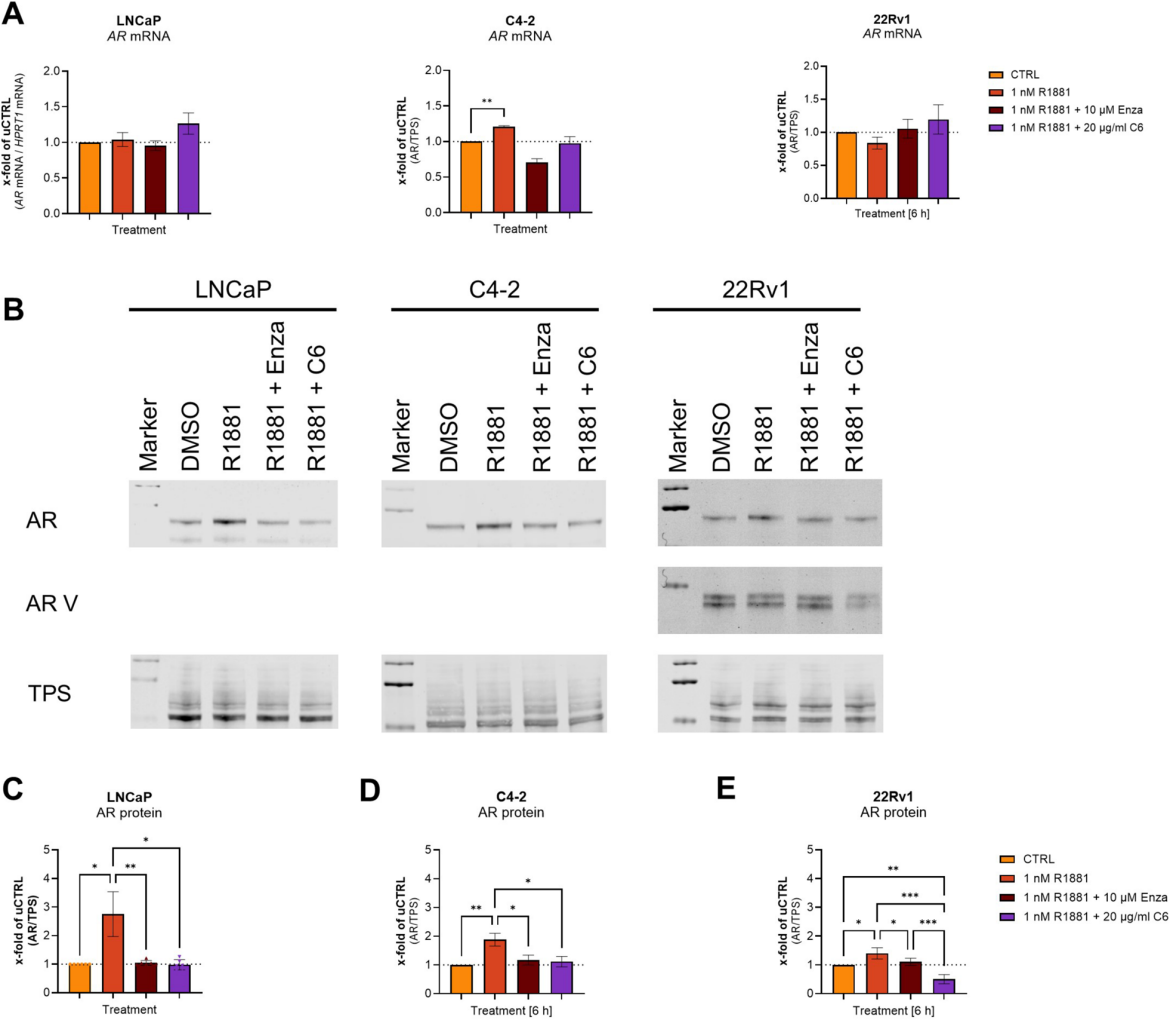
Translation, not transcription, is responsible for AR protein level changes after R1881 treatment. **A** Relative change of *AR* mRNA levels after 6 h treatment with DMSO (CTRL), 1 µM 1 nM R1881, 1 nM R1881 + 10 µM Enzalutamide, and 1 nM R1881 + 20 µg/ml Cycloheximide in LNCaP, C4-2 and 22Rv1 cells. Relative expression levels after treatment were shown as mean ± SEM of five independent experiments. **B** Representative western blots of the androgen receptor (AR), AR splice variant 7 (V7), and total protein stain (TPS) after treatment with DMSO (CTRL), 1 nM R1881, 1 nM R1881 + 10 µM Enzalutamide, and 1 nM R1881 + 20 µg/ml Cycloheximide in LNCaP, C4-2 and 22Rv1 cells. Uncropped western blots are displayed in Supplementary Fig. 4A. **C**-**E** Densitometry of AR protein levels relative to TPS in LNCaP (**C**), C4-2 (**D**), and 22Rv1 (**E**) cells. Relative expression levels compared to CTRL after treatment were shown as mean ± SD of six independent experiments. All differences highlighted by asterisks were statistically significant (*: *p* ≤ 0.05; **: *p* ≤ 0.01; ***: *p* ≤ 0.001)

To assess the influence of translation on AR activity, 350,000 cells/well were seeded for 24 h, followed by a 24 h starvation step in RPMI1640 without FBS. Subsequently, the cells were treated with DMSO (CTRL), 1 nM R1881, 1 nM R1881 + 10 µM Enza, or 1 nM R1881 + 20 µg/ml C6 for 6 h. qPCR analysis showed no change in the *AR* mRNA levels after 6 h of treatment (Fig. 3A). Western blot analysis (Fig. 3B–E) of the treated cells revealed that R1881 treatment led to a significant AR protein increase of 2.7-fold in LNCaP cells (Fig. 3C), 1.8-fold increase in C4-2 cells (Fig. 3D), and 1.4-fold increase in 22Rv1 cells (Fig. 3E). The AR splice variants (AR V), including AR expressed in 22Rv1 cells, were not influenced by the R1881 treatment (Supplementary Fig. 4B). The R1881-induced increase in AR protein levels was prevented by Enza treatment in all cell lines. In line with this, treatment with an inhibitor of eukaryotic translation C6 prevented the R1881-induced increase in AR (Fig. 3B–E). Moreover, the treatment significantly reduced AR V in 22Rv1 cells (Supplementary Fig. 4B). As nuclear translocation is an essential step in the regulation of AR activity and may influence protein levels, the influence of Enza and C6 on R1881-induced AR translocation was investigated using western blot analysis after nuclear and cytoplasmic extraction (Supplementary Fig. 5A). Treatment of LNCaP cells with R1881 increased nuclear AR levels, whereas cytoplasmic AR levels were unaltered (Supplementary Fig. 4B and C). Treatment with Enza prevented the R1881-induced increase in nuclear AR. In contrast, C6 treatment did not prevent the R1881-induced increase in the nuclear AR. However, in contrast to R1881-treated cells, cytoplasmic AR levels decreased (Supplementary Fig. 5B and C). These results validated that changes in translational activity are regulated by changes in AR protein levels after R1881 or Enza treatment.

### Inhibition of the eIF4F complex prevents androgen receptor activity

**Fig. 4:**
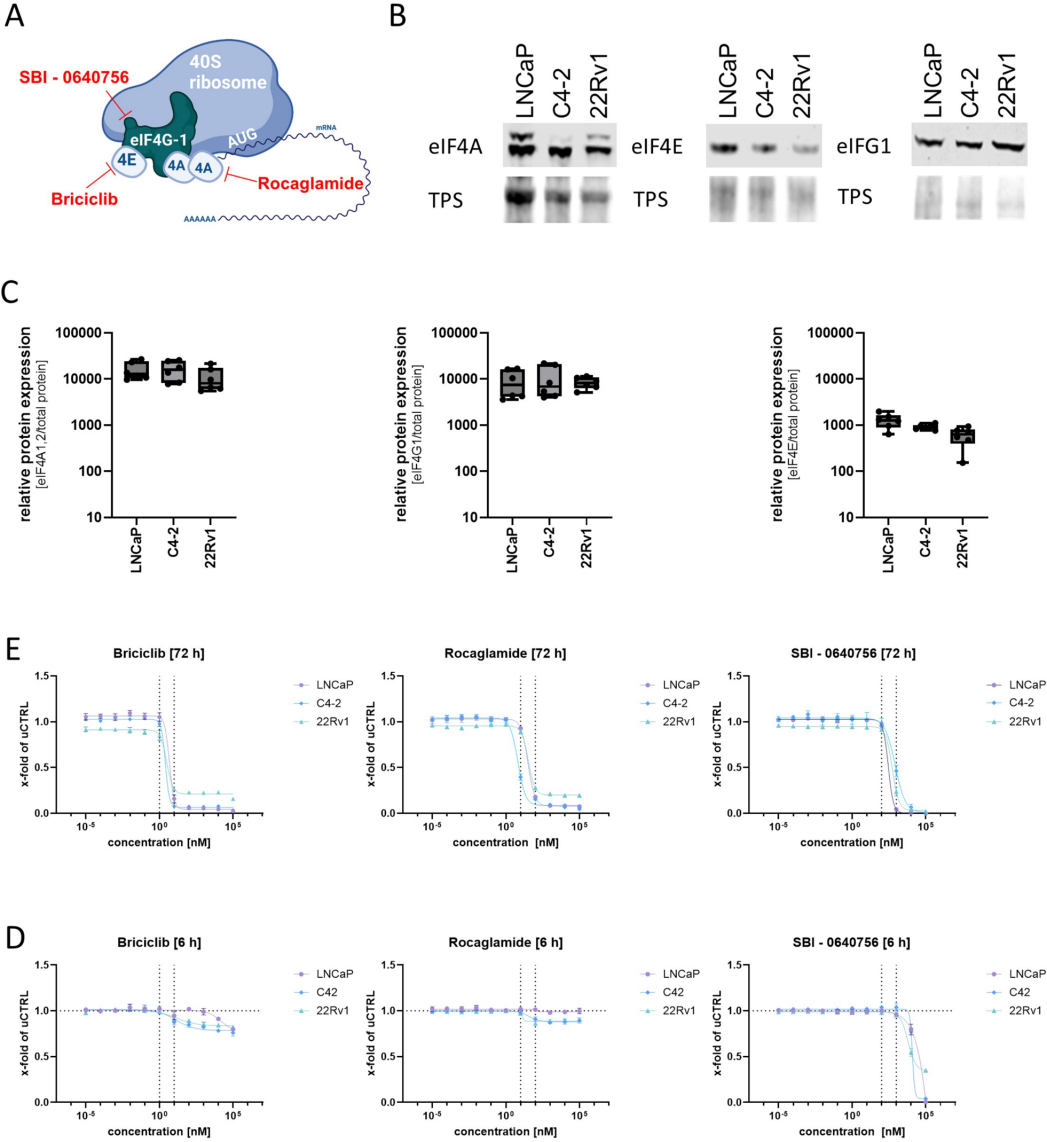
Influence of eiF4F inhibitors on PCa cells**.** **A** Schematic representation of the therapeutic target structures of Briciclib (eIF4E-inhibitor), Rocaglamide (Roca, eiF4A-inhibitor), and SBI-0640756 (eIF4G1-inhibitor) on the Eif4F complex. Created By Biorender. **B** Representative Western Blot of eiF4A, eiF4E, eiFG1, and total protein stain (TPS) of LNCaP, C4-2, 22Rv1 cells. Uncropped western blots are displayed in Supplementary Fig. 6, **C** Densitometry of eiF4A, eiF4E, and eiFG1 relative to TPS. Data is shown as box and whisker (min to max) of five independent western blot experiments. **D** Dose–response curves of change in cell proliferation after treatment with different concentrations of Briciclib, Rocaglamide, and SBI-0640756 of LNCaP, C4-2, 22Rv1 cells after 72 h. Data was plotted as mean ± SEM of the three biological replicates. **E** Dose–response curves of change in cell proliferation after treatment with different concentrations of Briciclib, Rocaglamide, and SBI-0640756 of LNCaP, C4-2, 22Rv1 cells after 6 h. Data was plotted as mean ± SEM of the three biological replicates

Previous studies have connected the AR to regulate the eukaryotic initiation factor 4F complex (eIF4F), a complex responsible for recruiting the 40S ribosomal subunit to the 5' cap of mRNAs during cap-dependent translation initiation [39, 40]. Several inhibitors of the subunits of the complex have been developed, including Briciclib (eIF4E-inhibitor), Rocaglamide (Roca, eiF4A-inhibitor), and SBI-0640756 (eIF4G1-inhibitor) which were used in this study (Fig. 4A). Western blot analysis revealed that all the targets of these inhibitors were highly expressed in the selected PCa cell models (Fig. 4B and C). To establish a working concentration for Briciclib, Roca, and SBI-0640756, dose–response experiments were performed for 72 h using LNCaP, C4-2, and 22Rv1 cells (Fig. 4D). The calculated IC_50_ values (Table 1) showed that Briciclib had the strongest, Roca the second strongest, and SBI-0640756 the weakest effect on cell growth of all cells examined.

**Table 1:** IC_50_ Values of eIF4F complex inhibitors

	Briciclib [nM]	Rocaglamid [nM]	SBI–0640756 [nM]
LNCaP	5.6	32	295
C4-2	2.8	6.7	847
22Rv1	3.3	35	542
Range	1–10	10–100	100–1000

**Fig. 5:**
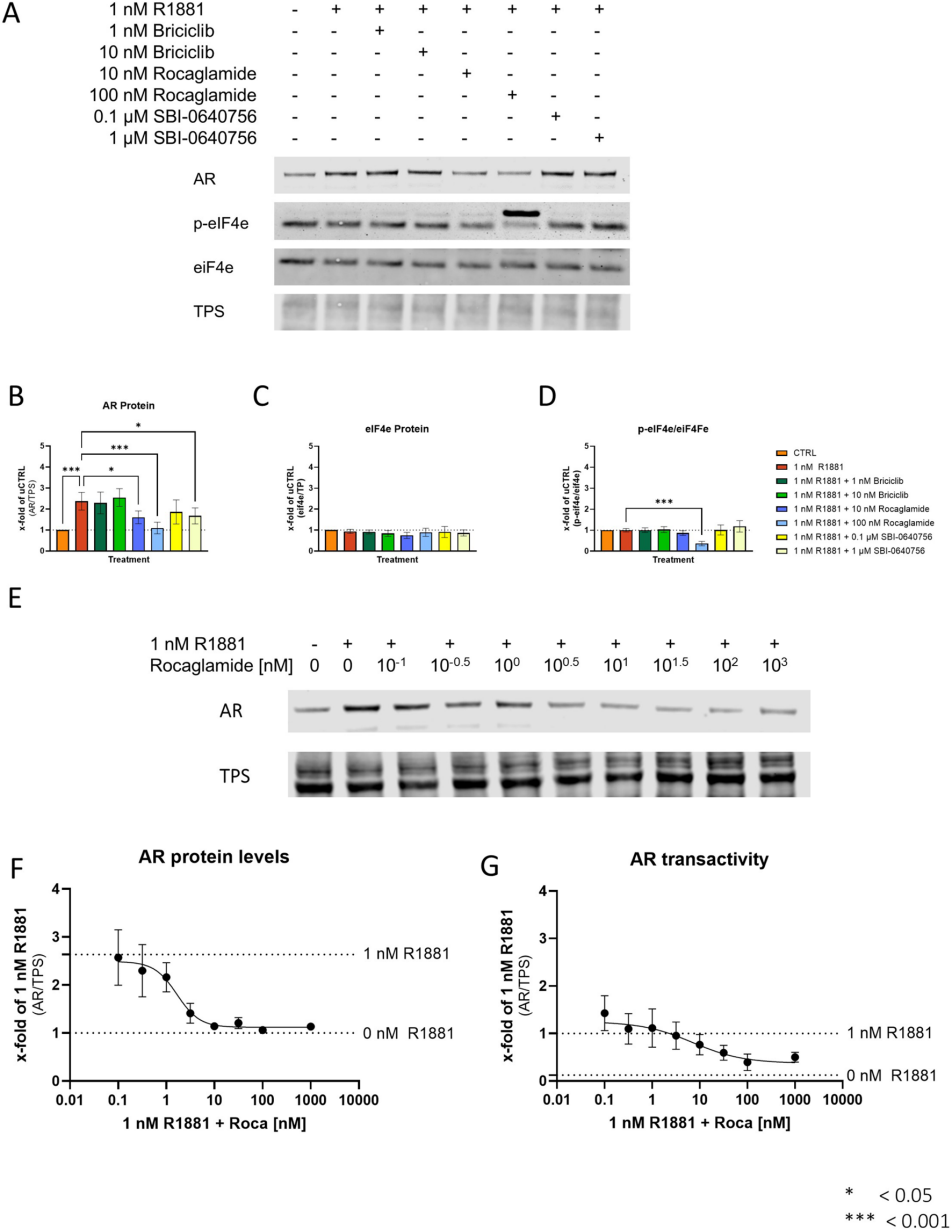
Influence of different concentrations of Rocaglamide on AR protein levels and transactivity in LNCaP cells. **A** Representative western blot to investigate the influence of different concentrations of Briciclib (eIF4E-inhibitor), Rocaglamide (Roca, eiF4A-inhibitor), and SBI-0640756 (eIF4G1-inhibitor) on the influence of R1881-induced increase of AR protein. In addition, p-eIF4E and eiF4E were investigated to assess the changes in eiF4F activity. Uncropped western blots are displayed in Supplementary Fig. 7. **B** Densitometry of AR protein levels relative to TPS. Relative expression levels compared to CTRL after treatment were shown as mean ± SD of five independent experiments. All differences highlighted by asterisks were statistically significant (*p ≤ 0.05; **p ≤ 0.01; ***p ≤ 0.001). **C** Densitometry of eiF4E protein levels relative to TPS. Relative expression levels compared to CTRL after treatment were shown as mean ± SD of five independent experiments. Relative expression levels after treatment were shown as mean ± SD of five independent experiments. **D** Densitometry of p-eiF4E protein levels relative to eiF4E. Relative expression levels compared to CTRL after treatment were shown as mean ± SD of five independent experiments. All differences highlighted by asterisks were statistically significant (*p ≤ 0.05; **p ≤ 0.01; ***p ≤ 0.001). **E** Representative western blot to investigate the influence of different concentrations of Rocaglamide on the influence of R1881-induced increase of AR protein. **F** Densitometry dose–response curves of AR protein levels relative to TPS. Relative expression levels compared to CTRL after treatment were shown as mean ± SD of five independent experiments. **G** Relative changes of AR transactivity to determine the influence of different concentrations of Rocaglamide on the influence of R1881-induced increase of AR transactivity. Changes were assessed by changes in *KLK3* normalised to *HPRT1* mRNA determined by qPCR. Relative expression levels after treatment were shown as mean ± SEM of five independent experiments

Based on the dose–response experiments, the concentrations 0.001 µM and 0.01 µM Briciclib, 0.01 µM and 0.1 µM Roca, and 1 µM and 10 µM SBI-0640756 were selected for further experiments, and their influence on the androgen regulation of the AR at the protein level was investigated. These concentrations also had negligible effects on cell number after 6 h, indicating the absence of apoptotic and necrotic events (Fig. 4E). To assess the influence of the chosen inhibitors on AR protein levels, 350,000 cells/well were seeded for 24 h, followed by a 24 h starvation step in RPMI1640 without FBS. Subsequently, the cells were treated with DMSO (CTRL), 1 nM R1881, 1 nM R1881 + Briciclib, Roca, or SBI-064075610 for 6 h. Western blot analysis (Fig. 5A) of the treated cells revealed that solely the two chosen concentrations of Roca significantly reduced the R1881-induced AR protein increase in LNCaP cells (Fig. 5B). To assess the influence of the inhibitors on the activity of the eIF4F complex, protein levels and phosphorylation of eIF4E were investigated, as eIF4E regulates the activity of the eIF4F complex [41]. Western blot analysis revealed no significant changes in eIF4E protein levels after treatment (Fig. 5C), whereas only Roca reduced eIF4E protein phosphorylation (Fig. 5D). Based on the influence of Roca on AR and p-eiF4E, Roca was selected for all further experiments.

To investigate whether this regulation is dose-dependent, the cells were treated with 1 nM R1881 and 1 nMR1881 + 0,1–1000 nM Roca (0.5 log_10_-steps) for 6 h. Western blot analysis revealed a dose–response relationship between Roca concentration and inhibition of the R1881-induced increase in AR protein levels (Fig. 5E and F). In addition, qPCR analysis revealed a dose–response relationship between Roca concentration and the inhibition of R1881-induced AR transactivity (Fig. 5G).

### Influence of the inhibition of the eukaryotic initiation factor 4F complex prevents increased androgen receptor protein levels and transactivity

**Fig. 6:**
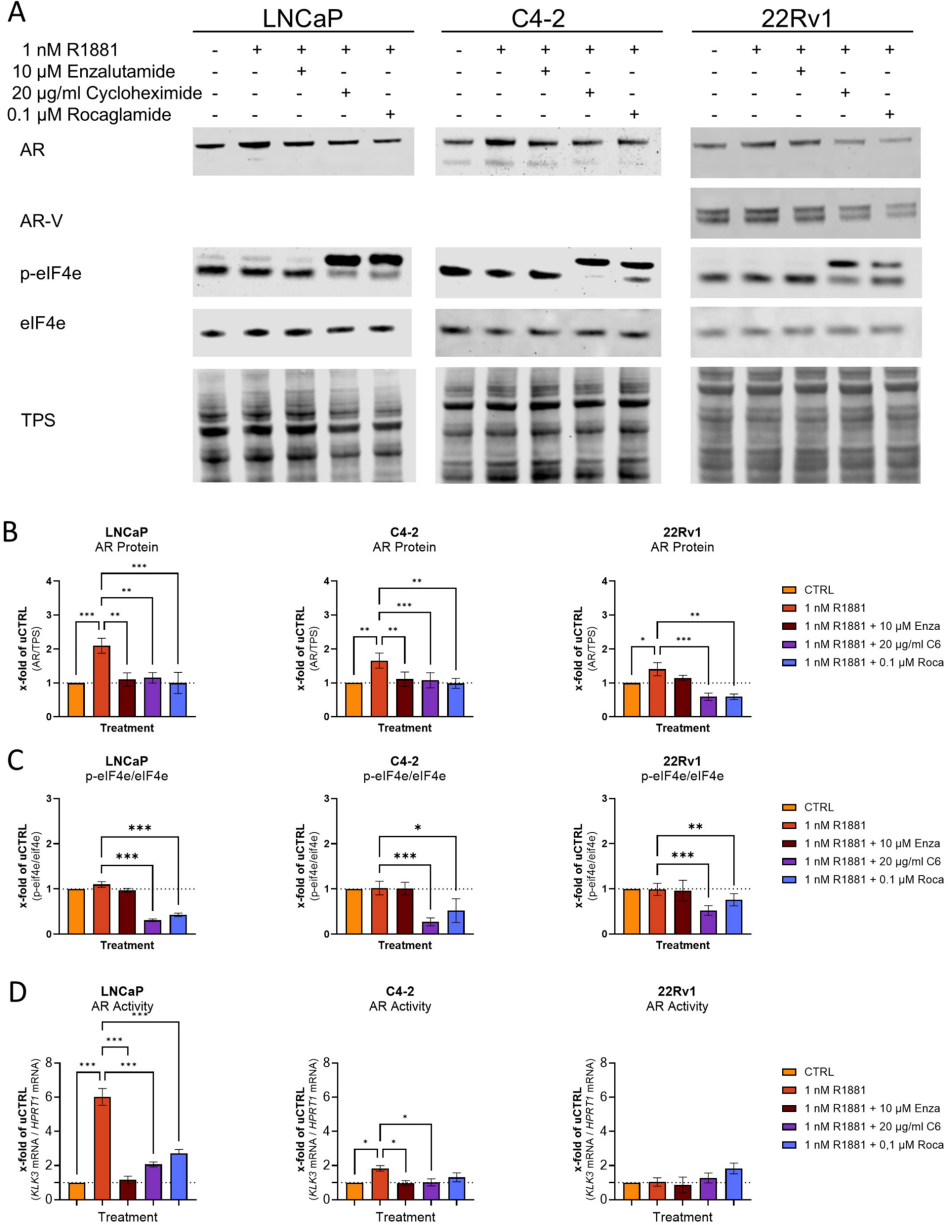
Rocaglamide reduces R1881-induced increase in AR protein levels and transactivity**.** **A** Representative western blot to investigate the influence of Rocaglamide on the R1881-induced increase of AR protein in LNCaP, C4-2, and 22RV1 cells. In addition, p-eIF4E and eiF4E were investigated to assess the changes in eiF4F activity. Uncropped Western blots are displayed in Fig. 8B. **B** Densitometry of AR protein levels relative to TPS in LNCaP, C4-2, and 22RV1 cells. Relative expression levels after treatment were shown as mean ± SD of six independent experiments. All differences highlighted by asterisks were statistically significant (*p ≤ 0.05; **p ≤ 0.01; ***p ≤ 0.001). **C** Densitometry of p-eiF4E protein levels relative to eiF4E in LNCaP, C4-2, and 22RV1 cells. Relative expression levels after treatment were shown as mean ± SD of five independent experiments. All differences highlighted by asterisks were statistically significant (*p ≤ 0.05; **p ≤ 0.01; ***p ≤ 0.001). **D** Relative changes of AR transactivity to determine the influence of Enzalutamide (Enza), Cycloheximide (C6), and Rocaglamide (Roca) on the influence of R1881-induced increase of AR transactivity. Changes were assessed by changes in *KLK3* normalised to *HPRT1* mRNA determined by qPCR. Relative expression levels after treatment were shown as mean ± SEM of six independent experiments. All differences highlighted by asterisks were statistically significant (**p ≤ 0.01; ***p ≤ 0.001)

To assess whether targeting eiF4F with Roca is a possible strategy for targeting AR signalling in HSPC and CRPC, R1881-induced AR activity was assessed. Western bot analysis revealed that in line with Enza and C6, it could prevent R1881-induced changes in AR and AR V proteins (Fig. 6A and B, Supplementary Fig. 8A). However, the analysis of p-eiF4E revealed that Enza did not reduce p-eiF4E levels, whereas C6 and Roca reduced p-eiF4E levels (Fig. 6C). As treatment with Enza, C6, and Roca prevented the R1881-induced increase in AR protein levels, the influence of the treatment on AR transactivity was investigated by measuring changes in the AR target gene *KLK3/PSA* (Fig. 6D). qPCR analysis (Fig. 6D) of the treated cells revealed that the R1881 treatment led to a significant AR transactivity increase of 6.0-fold in LNCaP and 1.8-fold in C4-2. In contrast, in 22Rv1 cells, AR transactivity did not change. In the HSPC cell line, LNCaP, Enza, C6, and Roca inhibited the R1881-induced increase in AR transactivity. However, neither C6 nor Roca could prevent AR transactivity as efficiently as Enza. The CRPC C4-2 cells, C6, and Roca, inhibited R1881-induced AR transactivity. In contrast, none of the treatments changed AR transactivity in the CRPC and Enza-resistant cell line 22Rv1.

## Discussion

AR is involved in the development of various diseases, including androgen insensitivity syndrome, spinal muscular atrophy, hypogonadism, and benign prostatic hyperplasia [42–45]. Moreover, AR is involved in the development and progression of PCa and is a key therapeutic target [3, 8]. Unfortunately, AR is also a linchpin for developing treatment resistance [14, 16, 46]. Various mechanisms have already been deciphered over the years, but the full extent of these mechanisms is not yet fully understood. Therefore, investigating new resistance mechanisms and therapeutic target structures around AR is still a translational and basic research topic [47, 48].

Several molecular adaptations have already been identified, including AR amplification and mutation, changes in co-regulator expression, and activation of bypass pathways [14, 16, 49–53]. Another underlying resistance mechanism and regulator of AR signalling appears to be increased AR protein stability [54, 55]. Heat shock proteins (HSP) and the proteasome system are reportedly involved in AR protein degradation [54–56]. Previous studies have revealed that high PIAS1 and STAT5 levels could enhance AR protein stability and mediate drug resistance [19, 54, 57]. High AR protein stability has also been linked to the castration-resistant cell line C4-2 after androgen withdrawal [58]. A study by Siciliano et al. concluded that there is a correlation between the level of AA-induced AR protein reduction and the AA response [17]. As this study aimed to investigate the role of AR protein changes in transactivity, the influence of proteasomes on Enza-induced AR reduction was examined. In line with previous observations, the use of different proteasome inhibitors could not reverse the effects of Enza on the AR protein [59]. This result indicates that, although the proteasome system plays an essential role in regulating the AR signalling pathway, it is not responsible for the Enza-induced reduction of the AR protein [55, 60]. One possible explanation is that MDM2-induced AR protein regulation seems to depend on the absence of androgens [55].

Proteomic analyses were performed, as proteasomal activity was not involved in AA-induced AR protein reduction in PCa cells. These studies showed that treatment with R1881 leads to an enrichment of proteins that alter translational activity and that Enza can prevent this enrichment. This observation aligns with previous studies linking the AR to translational regulation [40, 61, 62]. By using C6, it was also shown that inhibition of translation prevented R1881-induced AR protein level changes. In addition to the reduction in AR, the inhibition of translation also led to a reduction in the AR V proteins in 22Rv1. This variant is associated with resistance to therapy and poor disease progression [63–66]. These results, the previous observations by Siciliano et al*.*, and the short AR protein half-life time indicate that AR protein is strongly under translational regulation [12, 67]. Therefore, the fast regulation of the AR protein level by anti-androgens such as Enza may be due to the link between the AR and translation machinery.

As C6 has been reported to be highly toxic and mutagenic, its possible use as a therapeutic agent seems unreasonable [68]. Since proteome analysis showed that the eukaryotic translation initiation pathway was also affected by R1881 and there are already reports of a link between the AR and eiF4F complex, we investigated using eiF4F inhibitors whether the R1881-induced changes in the AR protein can be influenced [61]. Studies have suggested that the eIF4E subunit of the eIF4F complex enhances tumour growth and induces therapy resistance by increasing the translation of oncogene mRNAs [69]. Using the eiF4F inhibitor Roca, the effect on AR protein levels previously seen with C6 was reproduced. However, in an analysis of the eiF4F complex activity by changes in p-eiF4E, it could be revealed that Enza did not reduce eiF4F complex activity in contrast to C6 and Roca. This result indicates that Enza may reduce R1881's influence on translational pathways but does not directly inhibit the eiF4F complex. A possible mechanism is the regulation of the eiF4F complex by the translation repressor protein eukaryotic translation initiation factor 4E (eIF4E)-binding protein 1 (4E-BP1), which has been reported to be regulated by AR [70]. 4E-BP1 binds to eIF4E and inhibits the protein without altering its phosphorylation state [71]. However, further research is required to elucidate this mechanism.

The transactivity of steroid receptors such as AR is crucial for their function because it directly governs the receptor's ability to regulate gene expression, which is essential for mediating the physiological and pathological effects of steroid hormones, including the progression of PCa [5]. Therefore, we assessed the effect of translation inhibition on transactivity. These experiments revealed that the increase in AR transactivity after R1881 treatment was consistent with the increase in AR protein levels. Therefore, the hormone-sensitive LNCaP cells showed the most substantial increase in AR transactivity, the castration-resistant C4-2 cells showed only a weak increase in AR transactivity, and the castration-resistant and Enza-resistant 22Rv1 cells did not show any change in AR transactivity. Enza prevented this increase in LNCaP and C4-2 cells, whereas 22Rv1 was unaffected. This result reflects the reported status of the cell lines [18, 72]. Moreover, it confirms the hypothesis of Siciliano et al*.* that the change in AR protein after treatment is a surrogate for treatment response [17]. In line with the AR protein changes after treatment, the translation inhibitor C6 and the eiF4F inhibitor Roca reduced the R1881-induced increase in AR transactivation. However, the influence of these inhibitors on AR transactivation was lower than that of Enza. This reduced inhibition of R1881-induced AR transactivity can be attributed to the fact that Enza, in addition to inhibiting the increase in the AR protein, also prevents the transport of AR into the cell nucleus. Therefore, small amounts of AR can translocate further into the nucleus upon inhibition of translation after R1881 treatment, and thus bind to the DNA and trigger gene expression. In contrast to the results in LNCaP and C4-2 cells, no change in AR transactivity was observed because of the change in translational activity in 22Rv1. It can be assumed that intracellular molecular changes within the cell lead to AR playing a subordinate role and its role being taken over by other factors, such as the glucocorticoid receptor [72, 73] . Moreover, a crucial factor in which translational inhibition affects AR activity may be the upregulation of the AR protein, which is not the case for 22Rv1.

## Conclusion

**Fig. 7:**
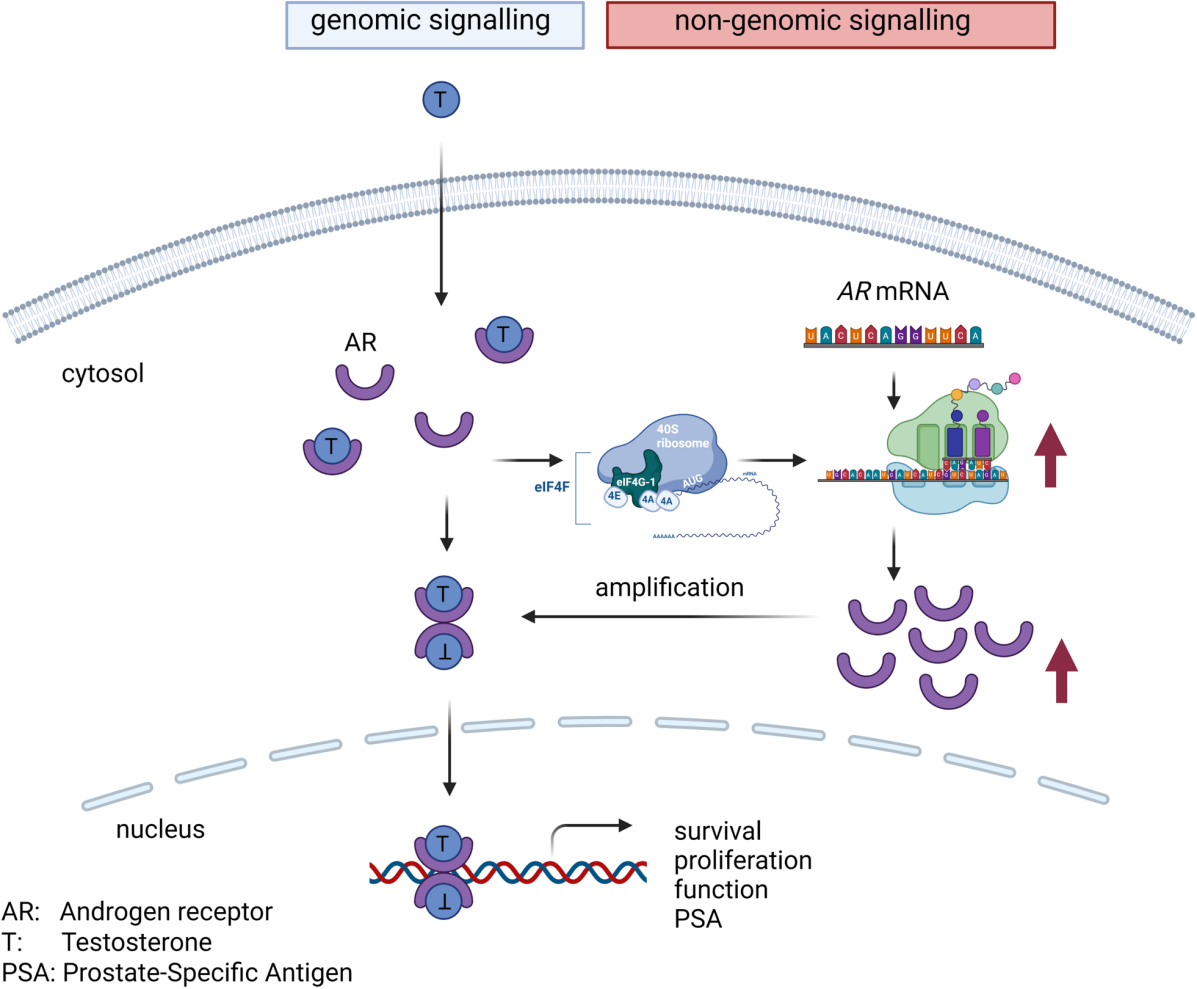
Graphical illustration of the role of translation in AR signalling. Created By Biorender

The study's findings suggest that the activation of the androgen receptor (AR) initiates a signalling cascade that rapidly enhances the AR protein levels by increasing the translation rate of AR (Fig. 7). This elevation in AR protein quantity augments AR transactivity, as the abundance of receptor proteins facilitates higher AR activity. Notably, in the used CRPC and Enza-resistant models, the effect on AR protein levels and transactivity was less pronounced. This observation leads to the hypothesis that the described mechanism might be impaired or dysregulated in advanced androgen receptor-reactive prostate cancer (PCa) cells. Consequently, future research should explore this hypothesis and whether the mechanism could serve as a therapeutic target to delay the progression of CRPC. The study's main limitation is the lack of proteomics data on the treatment at shorter time points, of the CRPC cell lines, and of post-translational modifications. Moreover, the treatment of ex vivo slide modes to investigate AR protein changes would be interesting.

## Supplementary Information

Supplementary Fig. 1: (A) Dose–response curves of change in cell proliferation after treatment with different concentrations of bortezomib, carfilzomib, epoxomicin, and (R)-MG-132 of LNCaP and LAPC4 after 72 h. Data was plotted as mean ± SEM of the three biological replicates (LNCaP cells) or one biological replicates (LAPC4). (E) Dose–response curves of change in cell proliferation after treatment with different concentrations of bortezomib, carfilzomib, epoxomicin, and (R)-MG-132 of LNCaP and LAPC4 after 6 h. Data was plotted as mean ± SEM of the three biological replicates (LNCaP cells) or one biological replicates (LAPC4). (C) Densitometric analysis and representative western blot of ubiquitin and GAPDH after treatment with bortezomib, carfilzomib, epoxomicin, and (R)-MG-132 for 6 h.

Supplementary Fig. 2: (A + B) Uncropped Western blots for Fig. 1A. (C + D) Volcano plot of differentially expressed proteins in LNCaP cells after treatment with 1 nM R1881 (C) and 1 nM R1881 + 10 µM Enzalutamide (D) for 6 h. Colour coding: grey = no statistically significant difference and not differentially expressed; blue = statistically significantly downregulated proteins; red = statistically significantly upregulated proteins. (E) Representative western blot to investigate the influence of cycloheximide (C6) on the proteome. (F) Denisometric analysis of the influence of C6 on the proteome and the analysis of protein concentration per cell after C6 for 6 h.

Supplementary Fig. 3: (A + B) Uncropped Western blots for Fig. 2B.

Supplementary Fig. 4: (A + B) Uncropped Western blots for Fig. 3B. (B) Densitometry of AR V protein levels relative to TPS in LNCaP, C4-2, and 22Rv1 cells. Relative expression levels after treatment were shown as mean ± SD of six independent experiments. All differences highlighted by asterisks were statistically significant (**: p ≤ 0.01; ***: p ≤ 0.001).

Supplementary Fig. 5: (A) Representative western blot after cytoplasmic and nuclear extraction to investigate the localisation of the AR after treatment with DMSO (CTRL), 1 nM R1881, 1 nM R1881 + 10 µM Enzalutamide, and 1 nM R1881 + 20 µg/ml Cycloheximide. (B) Densitometry of AR protein levels relative to TPS in the cytoplasm and nucleus of LNCaP. Relative expression levels after treatment were shown as mean ± SD of six independent experiments. All differences highlighted by asterisks were statistically significant (**: p ≤ 0.01; ***: p ≤ 0.001). (C) Percentage distribution of AR protein in the cytoplasmic and nuclear fractions.

Supplementary Fig. 6: (A + B) Uncropped Western blots for Fig. 4B.

Supplementary Fig. 7: (A) Uncropped Western blots for Fig. 5A. (B) Uncropped Western blots for Fig. 5E.

Supplementary Fig. 8: (A) Densitometry of AR V protein levels relative to TPS in LNCaP, C4-2, and 22Rv1 cells. Relative expression levels after treatment were shown as mean ± SD of six independent experiments. All differences highlighted by asterisks were statistically significant (**: p ≤ 0.01; ***: p ≤ 0.001). (B) Uncropped Western blots for Fig. 6A.

Supplementary Table 1: Chemicals and drugs used in the study. Supplementary Table 2: Antibodies used in this study. Supplementary Table 3: IC50 Values of the used proteasome inhibitors.

## Data Availability

The data presented in this study are openly available in https://github.com/justusisrael/ARTranslation at doi https://doi.org/10.5281/zenodo.13769868.

## Abbreviations

AR: Androgen receptor
PCa: Prostate cancer
CRPC: Castration-resistant prostate cancer
HSPC: Hormone-sensitive prostate cancer
BPH: Benign prostatic hyperplasia
HSP: Heat shock proteins
ARE: Androgen response element
Enza: Enzalutamide
AA: Anti-androgen
C6: Cycloheximide
Roca: Rocaglamide
eIF4F: Eukaryotic initiation factor 4F complex
AR V: Androgen receptor splice variants
4E-BP1: Eukaryotic translation initiation factor 4E (eIF4E)-binding protein 1
TPS: Total protein stain
GSEA: Gene set enrichment analysis

## References

[1] Claessens F, Denayer S, Van Tilborgh N, Kerkhofs S, Helsen C, Haelens A. Diverse roles of androgen receptor (AR) domains in AR-mediated signaling. Nucl Recept Signal. 2008;6: e008. 18612376 10.1621/nrs.06008 PMC2443950

[2] Jenster G, van der Korput HA, van Vroonhoven C, van der Kwast TH, Trapman J, Brinkmann AO. Domains of the human androgen receptor involved in steroid binding, transcriptional activation, and subcellular localisation. Mol Endocrinol. 1991;5(10):1396–404. 1775129 10.1210/mend-5-10-1396

[3] Crona DJ, Whang YE. Androgen receptor-dependent and -independent mechanisms involved in prostate cancer therapy resistance. Cancers. 2017;9(6):67. 28604629 10.3390/cancers9060067 PMC5483886

[4] Green SM, Mostaghel EA, Nelson PS. Androgen action and metabolism in prostate cancer. Mol Cell Endocrinol. 2012;360(1–2):3–13. 22453214 10.1016/j.mce.2011.09.046 PMC4124858

[5] Davey RA, Grossmann M. Androgen receptor structure, function and biology: from bench to bedside. Clin Biochem Rev. 2016;37(1):3–15. 27057074 PMC4810760

[6] Shukla GC, Plaga AR, Shankar E, Gupta S. Androgen receptor-related diseases: what do we know? Andrology. 2016;4(3):366–81. 26991422 10.1111/andr.12167

[7] Tirabassi G, Corona G, Biagioli A, Buldreghini E, delli Muti N, Maggi M, et al. Influence of androgen receptor CAG polymorphism on sexual function recovery after testosterone therapy in late-onset hypogonadism. J Sex Med. 2015;12(2):381–8. 25443437 10.1111/jsm.12790

[8] Watson PA, Arora VK, Sawyers CL. Emerging mechanisms of resistance to androgen receptor inhibitors in prostate cancer. Nat Rev Cancer. 2015;15(12):701–11. 26563462 10.1038/nrc4016 PMC4771416

[9] Nadal M, Prekovic S, Gallastegui N, Helsen C, Abella M, Zielinska K, et al. Structure of the homodimeric androgen receptor ligand-binding domain. Nat Commun. 2017;8:14388. 28165461 10.1038/ncomms14388 PMC5303882

[10] Culig Z, Puhr M. Androgen receptor-interacting proteins in prostate cancer development and therapy resistance. Am J Pathol. 2024;194(3):324–34.38104650 10.1016/j.ajpath.2023.12.003

[11] Kallio PJ, Poukka H, Moilanen A, Jänne OA, Palvimo JJ. Androgen receptor-mediated transcriptional regulation in the absence of direct interaction with a specific DNA element. Mol Endocrinol. 1995;9(8):1017–28. 7476976 10.1210/mend.9.8.7476976

[12] Siciliano T, Sommer U, Beier A-MK, Stope MB, Borkowetz A, Thomas C, et al. The androgen hormone-induced increase in androgen receptor protein expression is caused by the autoinduction of the androgen receptor translational activity. Curr Issues Mol Biol. 2022;44(2):597. 35723327 10.3390/cimb44020041 PMC8928990

[13] Bray F, Laversanne M, Sung H, Ferlay J, Siegel RL, Soerjomataram I, et al. Global cancer statistics 2022: GLOBOCAN estimates of incidence and mortality worldwide for 36 cancers in 185 countries. CA Cancer J Clin. 2024;74(3):229–63. 38572751 10.3322/caac.21834

[14] Santer FR, Erb HH, McNeill RV. Therapy escape mechanisms in the malignant prostate. Semin Cancer Biol. 2015;35:133–44. 26299608 10.1016/j.semcancer.2015.08.005

[15] Tilki D, van den Bergh RCN, Briers E, Van den Broeck T, Brunckhorst O, Darraugh J, et al. EAU-EANM-ESTRO-ESUR-ISUP-SIOG guidelines on prostate cancer. Part II-2024 update: Treatment of relapsing and metastatic prostate cancer. Eur Urol. 2024. 10.1016/j.eururo.2024.04.010 38688773

[16] Claessens F, Helsen C, Prekovic S, Van den Broeck T, Spans L, Van Poppel H, et al. Emerging mechanisms of enzalutamide resistance in prostate cancer. Nat Rev Urol. 2014;11(12):712–6. 25224448 10.1038/nrurol.2014.243

[17] Siciliano T, Simons IH, Beier A-MK, Ebersbach C, Aksoy C, Seed RI, et al. A systematic comparison of antiandrogens identifies androgen receptor protein stability as an indicator for treatment response. Life. 2021;11(9):874. 34575023 10.3390/life11090874 PMC8468615

[18] Thalmann GN, Sikes RA, Wu TT, Degeorges A, Chang SM, Ozen M, et al. LNCaP progression model of human prostate cancer: androgen-independence and osseous metastasis. Prostate. 2000;44(2):91–103. 10881018 10.1002/1097-0045(20000701)44:2<91::aid-pros1>3.0.co;2-l

[19] Erb HHH, Bodenbender J, Handle F, Diehl T, Donix L, Tsaur I, et al. Assessment of STAT5 as a potential therapy target in enzalutamide-resistant prostate cancer. PLoS ONE. 2020;15(8): e0237248. 32790723 10.1371/journal.pone.0237248 PMC7425943

[20] Beier AK, Ebersbach C, Siciliano T, Scholze J, Hofmann J, Hönscheid P, et al. Targeting the glutamine metabolism to suppress cell proliferation in mesenchymal docetaxel-resistant prostate cancer. Oncogene. 2024. 10.1038/s41388-024-03059-4 PMC11196217 38750263

[21] Rao X, Huang X, Zhou Z, Lin X. An improvement of the 2ˆ(-delta delta CT) method for quantitative real-time polymerase chain reaction data analysis. Biostat Bioinforma Biomath. 2013;3(3):71–85. 25558171 PMC4280562

[22] Livak KJ, Schmittgen TD. Analysis of relative gene expression data using real-time quantitative PCR and the 2(-Delta Delta C(T)) Method. Methods. 2001;25(4):402–8. 11846609 10.1006/meth.2001.1262

[23] Seed RI, Taurozzi AJ, Wilcock DJ, Nappo G, Erb HHH, Read ML, et al. The putative tumour suppressor protein Latexin is secreted by prostate luminal cells and is downregulated in malignancy. Sci Rep. 2019;9(1):5120. 30914656 10.1038/s41598-019-41379-8 PMC6435711

[24] Küster JHS, Erb HHH, Ahrend H, Abazid A, Stope MB. Modulation of the prostate cancer resistance factor Hsp27 by the chemotherapeutic drugs abiraterone, Cabazitaxel Docetaxel and Enzalutamide. Anticancer Res. 2024;44(7):2815–21. 38925843 10.21873/anticanres.17093

[25] Müller H, Lesur A, Dittmar G, Gentzel M, Kettner K. Proteomic consequences of TDA1 deficiency in Saccharomyces cerevisiae: protein kinase Tda1 is essential for Hxk1 and Hxk2 serine 15 phosphorylation. Sci Rep. 2022;12(1):18084. 36302925 10.1038/s41598-022-21414-x PMC9613766

[26] Wickham H, François R. dplyr: A Grammar of Data Manipulation 2014.

[27] Team R. A language and environment for statistical computing. Computing. 2006;1.

[28] Okoye K, Hosseini S. Introduction to R programming and RStudio integrated development environment (IDE). 2024. p. 3–24.

[29] Stekhoven DJ, Buhlmann P. MissForest–non-parametric missing value imputation for mixed-type data. Bioinformatics. 2012;28(1):112–8. 22039212 10.1093/bioinformatics/btr597

[30] Ritchie ME, Phipson B, Wu D, Hu Y, Law CW, Shi W, et al. limma powers differential expression analyses for RNA-sequencing and microarray studies. Nucleic Acids Res. 2015;43(7): e47. 25605792 10.1093/nar/gkv007 PMC4402510

[31] Wu T, Hu E, Xu S, Chen M, Guo P, Dai Z, et al. clusterProfiler 4.0: a universal enrichment tool for interpreting omics data. Innovtion (Camb). 2021;2(3): 100141. 10.1016/j.xinn.2021.100141 PMC8454663 34557778

[32] Yu G, Wang LG, Han Y, He QY. clusterProfiler: an R package for comparing biological themes among gene clusters. OMICS. 2012;16(5):284–7. 22455463 10.1089/omi.2011.0118 PMC3339379

[33] Yu G, He QY. ReactomePA: an R/Bioconductor package for reactome pathway analysis and visualisation. Mol Biosyst. 2016;12(2):477–9. 26661513 10.1039/c5mb00663e

[34] Yu G, Wang LG, Yan GR, He QY. DOSE: an R/Bioconductor package for disease ontology semantic and enrichment analysis. Bioinformatics. 2015;31(4):608–9. 25677125 10.1093/bioinformatics/btu684

[35] Gu Z. Complex heatmap visualisation. Imeta. 2022;1(3): e43. 38868715 10.1002/imt2.43 PMC10989952

[36] Gu Z, Eils R, Schlesner M. Complex heatmaps reveal patterns and correlations in multidimensional genomic data. Bioinformatics. 2016;32(18):2847–9. 27207943 10.1093/bioinformatics/btw313

[37] Gu Z, Gu L, Eils R, Schlesner M, Brors B. circlize Implements and enhances circular visualisation in R. Bioinformatics. 2014;30(19):2811–2. 24930139 10.1093/bioinformatics/btu393

[38] Lee DK, Chang C. Expression and degradation of androgen receptor: mechanism and clinical implication. J Clin Endocrinol Metab. 2003;88(9):4043–54. 12970260 10.1210/jc.2003-030261

[39] Jana S. The androgen receptor regulates a druggable translational regulon in advanced prostate cancer. Science Translational Medicine. 2019. 10.1126/scitranslmed.aaw4993 PMC6746573 31366581

[40] Stone L. AR — the link between transcription and translation. Nat Rev Urol. 2019;16(10):565. 31455866 10.1038/s41585-019-0229-8

[41] Piserà A, Campo A, Campo S. Structure and functions of the translation initiation factor eIF4E and its role in cancer development and treatment. J Genet Genomics. 2018;45(1):13–24. 29396141 10.1016/j.jgg.2018.01.003

[42] Brown TR. Human androgen insensitivity syndrome. J Androl. 1995;16(4):299–303. 8537246

[43] McEwan IJ. Structural and functional alterations in the androgen receptor in spinal bulbar muscular atrophy. Biochem Soc Trans. 2001;29(Pt 2):222–7. 11356158 10.1042/0300-5127:0290222

[44] Kumar P, Kumar N, Thakur DS, Patidar A. Male hypogonadism: symptoms and treatment. J Adv Pharm Technol Res. 2010;1(3):297–301. 22247861 10.4103/0110-5558.72420 PMC3255409

[45] Webber R. Benign prostatic hyperplasia. Clin Evid. 2006;15:1213–26. 16973049

[46] Handle F, Prekovic S, Helsen C, Van den Broeck T, Smeets E, Moris L, et al. Drivers of AR indifferent anti-androgen resistance in prostate cancer cells. Sci Rep. 2019;9(1):13786. 31551480 10.1038/s41598-019-50220-1 PMC6760229

[47] Mehralivand S, Thomas C, Puhr M, Claessens F, van de Merbel AF, Dubrovska A, et al. New advances of the androgen receptor in prostate cancer: report from the 1st international androgen receptor symposium. J Transl Med. 2024;22(1):71. 38238739 10.1186/s12967-024-04878-5 PMC10795409

[48] Israel JS, Marcelin LM, Thomas C, Szczyrbova E, Fuessel S, Puhr M, et al. Emerging frontiers in androgen receptor research for prostate cancer: insights from the 2nd international androgen receptor symposium. J Exp Clin Cancer Res. 2024;43(1):194. 39014480 10.1186/s13046-024-03125-5 PMC11253403

[49] Koivisto P, Kononen J, Palmberg C, Tammela T, Hyytinen E, Isola J, et al. Androgen receptor gene amplification: a possible molecular mechanism for androgen deprivation therapy failure in prostate cancer. Can Res. 1997;57(2):314–9. 9000575

[50] Heinlein CA, Chang C. Androgen receptor in prostate cancer. Endocr Rev. 2004;25(2):276–308. 15082523 10.1210/er.2002-0032

[51] Culig Z. Molecular mechanisms of enzalutamide resistance in prostate cancer. Curr Mol Biol Rep. 2017;3(4):230–5. 29214142 10.1007/s40610-017-0079-1 PMC5700216

[52] King CJ, Woodward J, Schwartzman J, Coleman DJ, Lisac R, Wang NJ, et al. Integrative molecular network analysis identifies emergent enzalutamide resistance mechanisms in prostate cancer. Oncotarget. 2017;8(67):111084–95. 29340039 10.18632/oncotarget.22560 PMC5762307

[53] Tan SH, Dagvadorj A, Shen F, Gu L, Liao Z, Abdulghani J, et al. Transcription factor Stat5 synergises with androgen receptor in prostate cancer cells. Can Res. 2008;68(1):236–48. 10.1158/0008-5472.CAN-07-2972 18172316

[54] Lakshmana G, Baniahmad A. Interference with the androgen receptor protein stability in therapy-resistant prostate cancer. Int J Cancer. 2019;144(8):1775–9. 30125354 10.1002/ijc.31818

[55] Khatiwada P, Rimal U, Han Z, Shemshedini L. MDM2 regulates the stability of AR, AR-V7, and TM4SF3 proteins in prostate cancer. Endocr Oncol. 2024;4(1): e230017. 38410785 10.1530/EO-23-0017 PMC10895308

[56] Liu C, Lou W, Yang JC, Liu L, Armstrong CM, Lombard AP, et al. Proteostasis by STUB1/HSP70 complex controls sensitivity to androgen receptor targeted therapy in advanced prostate cancer. Nat Commun. 2018;9(1):4700. 30446660 10.1038/s41467-018-07178-x PMC6240084

[57] Thomas C, Zoubeidi A, Kuruma H, Fazli L, Lamoureux F, Beraldi E, et al. Transcription factor Stat5 knockdown enhances androgen receptor degradation and delays castration-resistant prostate cancer progression in vivo. Mol Cancer Ther. 2011;10(2):347–59. 21216933 10.1158/1535-7163.MCT-10-0850

[58] Wang J, Zhang H, Zhang X, Wang P, Wang H, Huang F, et al. PC-1 works in conjunction with E3 ligase CHIP to regulate androgen receptor stability and activity. Oncotarget. 2016;7(49):81377–88. 27835608 10.18632/oncotarget.13230 PMC5348399

[59] Erb HHH, Oster MA, Gelbrich N, Cammann C, Thomas C, Mustea A, et al. Enzalutamide-induced proteolytic degradation of the androgen receptor in prostate cancer cells is mediated only to a limited extent by the proteasome system. Anticancer Res. 2021;41(7):3271–9. 34230121 10.21873/anticanres.15113

[60] Vummidi Giridhar P, Williams K, VonHandorf AP, Deford PL, Kasper S. Constant degradation of the androgen receptor by MDM2 conserves prostate cancer stem cell integrity. Can Res. 2019;79(6):1124–37. 10.1158/0008-5472.CAN-18-1753 PMC6428062 30626627

[61] Liu Y, Horn JL, Banda K, Goodman AZ, Lim Y, Jana S, et al. The androgen receptor regulates a druggable translational regulon in advanced prostate cancer. Sci Transl Med. 2019;11(503). 10.1126/scitranslmed.aaw4993 PMC6746573 31366581

[62] Overcash RF, Chappell VA, Green T, Geyer CB, Asch AS, Ruiz-Echevarria MJ. Androgen signaling promotes translation of TMEFF2 in prostate cancer cells via phosphorylation of the alpha subunit of the translation initiation factor 2. PLoS ONE. 2013;8(2): e55257. 23405127 10.1371/journal.pone.0055257 PMC3566213

[63] Wüstmann N, Seitzer K, Humberg V, Vieler J, Grundmann N, Steinestel J, et al. Co-expression and clinical utility of AR-FL and AR splice variants AR-V3, AR-V7 and AR-V9 in prostate cancer. Biomark Res. 2023;11(1):37. 37016463 10.1186/s40364-023-00481-w PMC10074820

[64] Schlack K, Seitzer K, Wüstmann N, Humberg V, Grundmann N, Steinestel J, et al. Comparison of circulating tumor cells and AR-V7 as clinical biomarker in metastatic castration-resistant prostate cancer patients. Sci Rep. 2022;12(1):11846. 35831403 10.1038/s41598-022-16094-6 PMC9279395

[65] Erb HHH, Sparwasser P, Diehl T, Hemmerlein-Thomas M, Tsaur I, Jüngel E, et al. AR-V7 protein expression in circulating tumour cells is not predictive of treatment response in mCRPC. Urol Int. 2020;104(3–4):253–62. 31955172 10.1159/000504416

[66] Qu Y, Dai B, Ye D, Kong Y, Chang K, Jia Z, et al. Constitutively active AR-V7 plays an essential role in the development and progression of castration-resistant prostate cancer. Sci Rep. 2015;5:7654. 25563505 10.1038/srep07654 PMC4288210

[67] Harada N, Murata Y, Yamaji R, Miura T, Inui H, Nakano Y. Resveratrol down-regulates the androgen receptor at the post-translational level in prostate cancer cells. J Nutr Sci Vitaminol (Tokyo). 2007;53(6):556–60. 18202547 10.3177/jnsv.53.556

[68] Lawana V, Korrapati MC, Mehendale HM. Cycloheximide. In: Wexler P, editor. Encyclopedia of Toxicology. 3rd ed. Oxford: Academic Press; 2014. p. 1103–5.

[69] D’Abronzo LS, Ghosh PM. eIF4E phosphorylation in prostate cancer. Neoplasia. 2018;20(6):563–73. 29730477 10.1016/j.neo.2018.04.003 PMC5994774

[70] Mirzakhani K, Baniahmad A. Protein translation controlled by the androgen receptor in prostate cancer: a novel therapeutic option? Transl Cancer Res. 2020;9(4):2171–4. 35117576 10.21037/tcr.2020.02.31 PMC8797708

[71] Qin X, Jiang B, Zhang Y. 4E-BP1, a multifactor regulated multifunctional protein. Cell Cycle. 2016;15(6):781–6. 26901143 10.1080/15384101.2016.1151581 PMC4845917

[72] Smith R, Liu M, Liby T, Bayani N, Bucher E, Chiotti K, et al. Enzalutamide response in a panel of prostate cancer cell lines reveals a role for glucocorticoid receptor in enzalutamide resistant disease. Sci Rep. 2020;10(1):21750. 33303959 10.1038/s41598-020-78798-x PMC7729982

[73] Arora VK, Schenkein E, Murali R, Subudhi SK, Wongvipat J, Balbas MD, et al. Glucocorticoid receptor confers resistance to anti-androgens by bypassing androgen receptor blockade. Cell. 2013;155(6):1309–22. 24315100 10.1016/j.cell.2013.11.012 PMC3932525

