# Supplementary figures and images for: The impact of androgen-induced translation in modulating androgen receptor activity
Source: Biol Direct. 2024 Nov 11;19:111. doi: 10.1186/s13062-024-00550-6 (PMC11555926; doi:10.1186/s13062-024-00550-6)

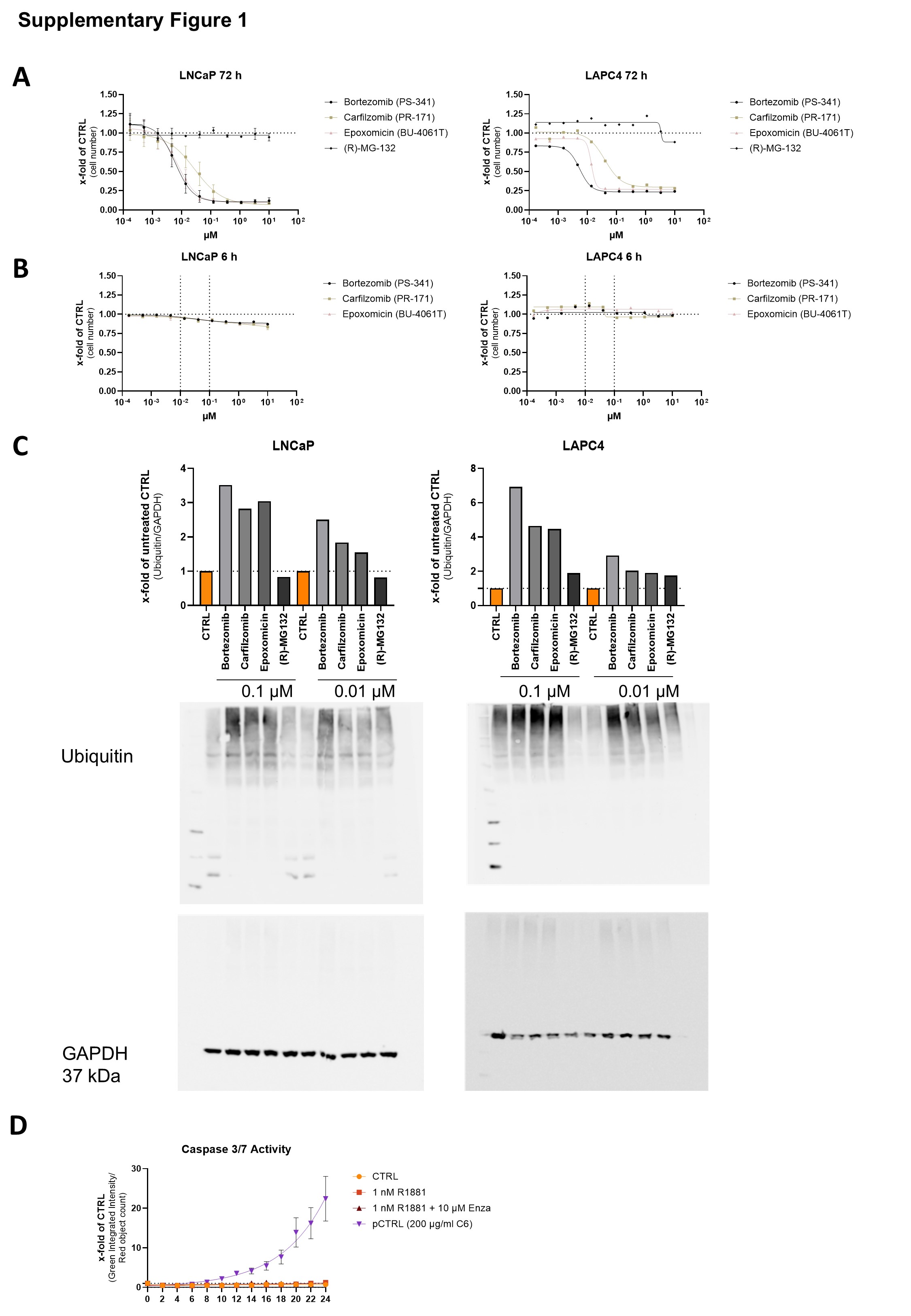

Supplement: Supplementary file 1 — Supplementary Fig. 1: (A) Dose–response curves of change in cell proliferation after treatment with different concentrations of bortezomib, carfilzomib, epoxomicin, and (R)-MG-132 of LNCaP and LAPC4 after 72 h. Data was plotted as mean ± SEM of the three biological replicates (LNCaP cells) or one biological replicates (LAPC4). (E) Dose–response curves of change in cell proliferation after treatment with different concentrations of bortezomib, carfilzomib, epoxomicin, and (R)-MG-132 of LNCaP and LAPC4 after 6 h. Data was plotted as mean ± SEM of the three biological replicates (LNCaP cells) or one biological replicates (LAPC4). (C) Densitometric analysis and representative western blot of ubiquitin and GAPDH after treatment with bortezomib, carfilzomib, epoxomicin, and (R)-MG-132 for 6 h. [file 13062_2024_550_MOESM1_ESM.jpg]

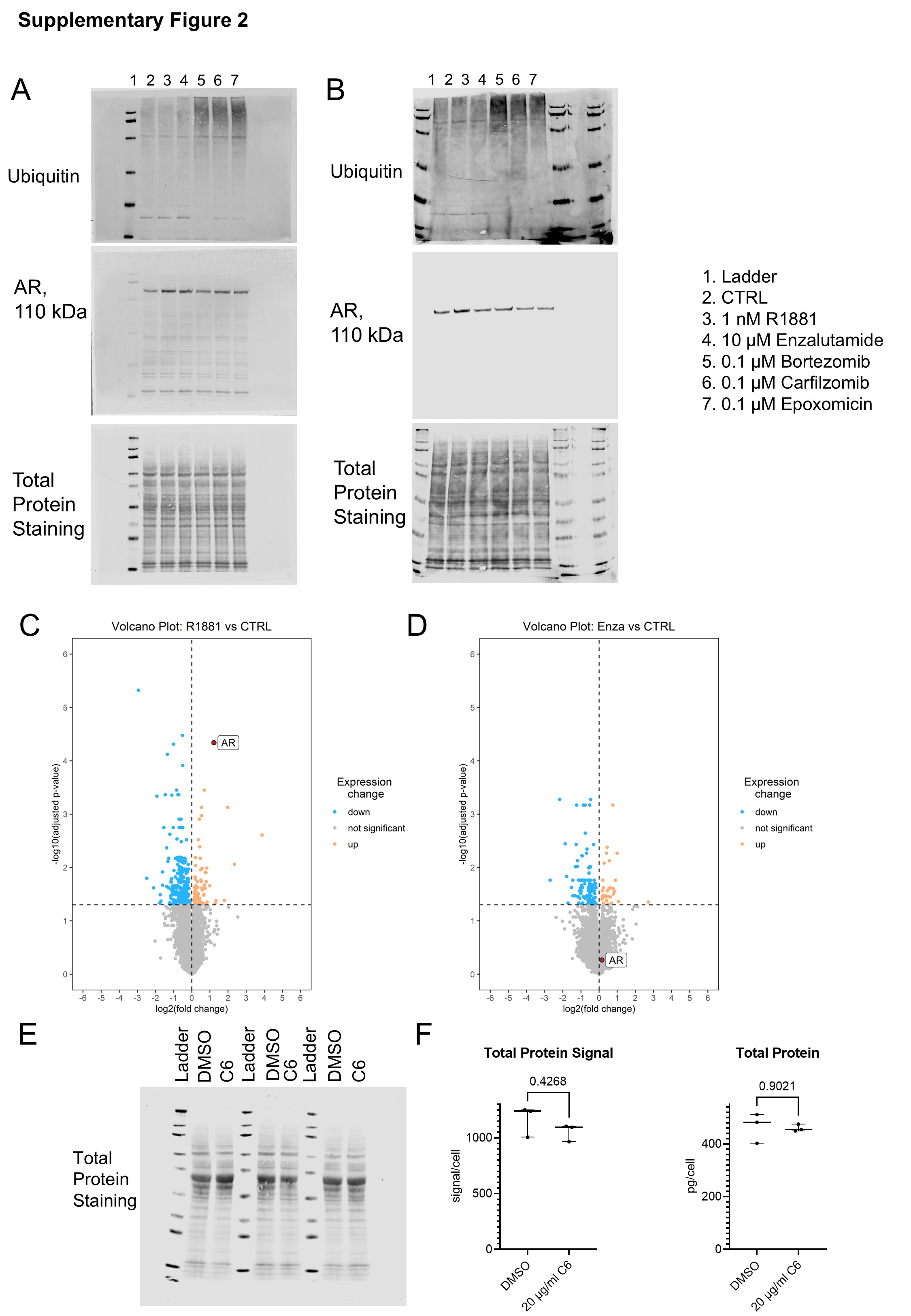

Supplement: Supplementary file 2 — Supplementary Fig. 2: (A + B) Uncropped Western blots for Fig. 1A. (C + D) Volcano plot of differentially expressed proteins in LNCaP cells after treatment with 1 nM R1881 (C) and 1 nM R1881 + 10 µM Enzalutamide (D) for 6 h. Colour coding: grey = no statistically significant difference and not differentially expressed; blue = statistically significantly downregulated proteins; red = statistically significantly upregulated proteins. (E) Representative western blot to investigate the influence of cycloheximide (C6) on the proteome. (F) Denisometric analysis of the influence of C6 on the proteome and the analysis of protein concentration per cell after C6 for 6 h. [file 13062_2024_550_MOESM2_ESM.jpg]

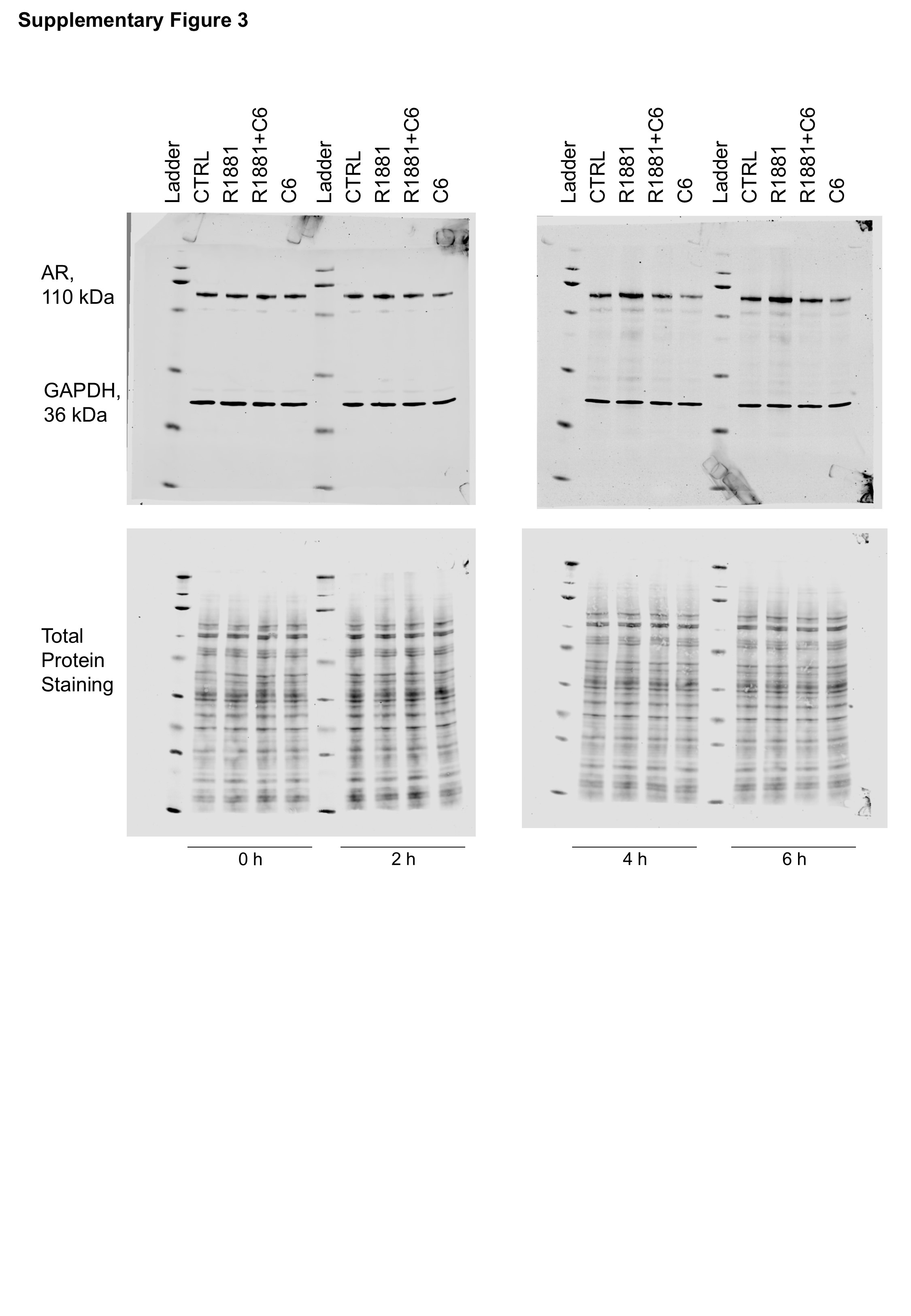

Supplement: Supplementary file 3 — Supplementary Fig. 3: (A + B) Uncropped Western blots for Fig. 2B. [file 13062_2024_550_MOESM3_ESM.jpg]

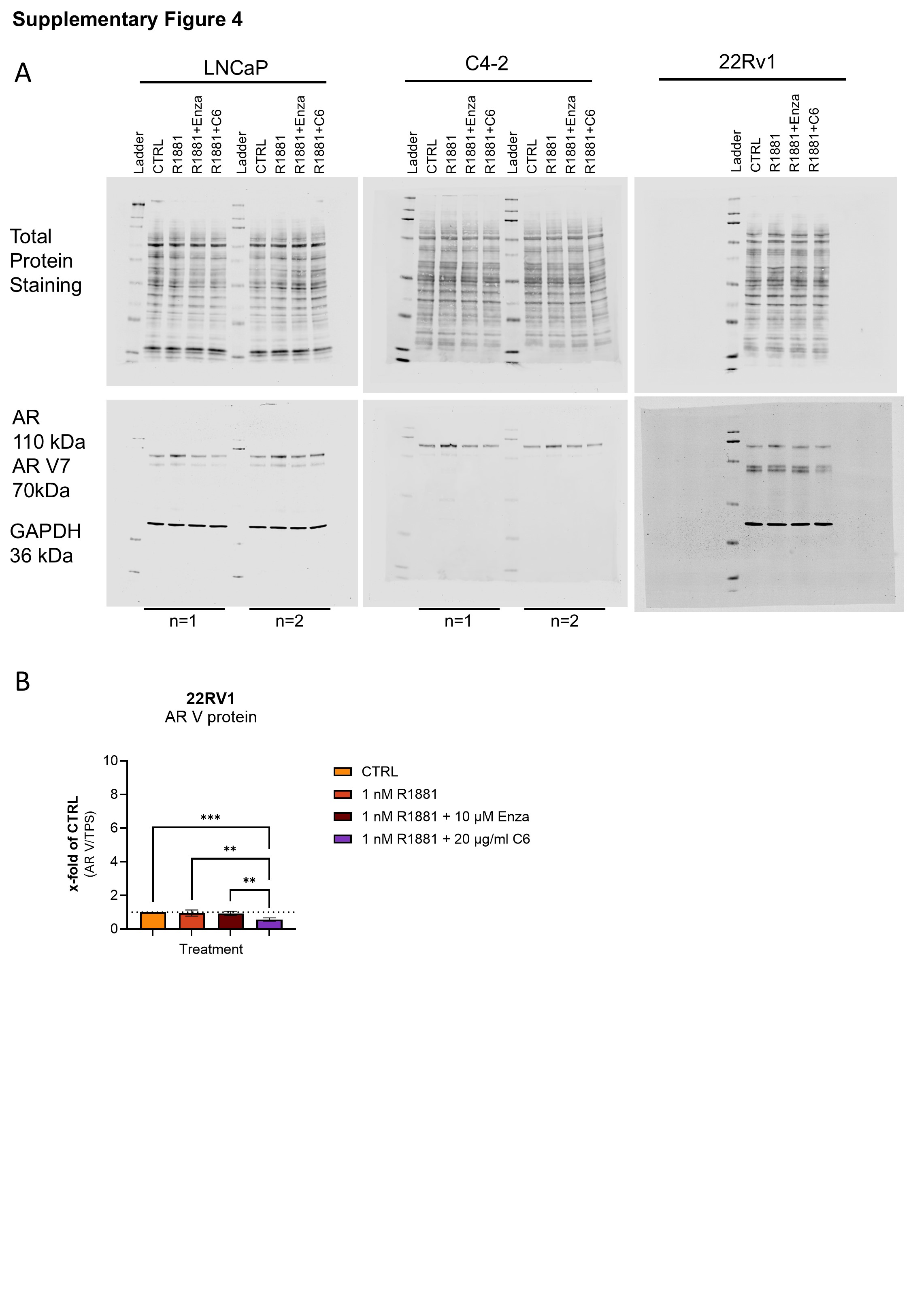

Supplement: Supplementary file 4 — Supplementary Fig. 4: (A + B) Uncropped Western blots for Fig. 3B. (B) Densitometry of AR V protein levels relative to TPS in LNCaP, C4-2, and 22Rv1 cells. Relative expression levels after treatment were shown as mean ± SD of six independent experiments. All differences highlighted by asterisks were statistically significant (**: p ≤ 0.01; ***: p ≤ 0.001). [file 13062_2024_550_MOESM4_ESM.jpg]

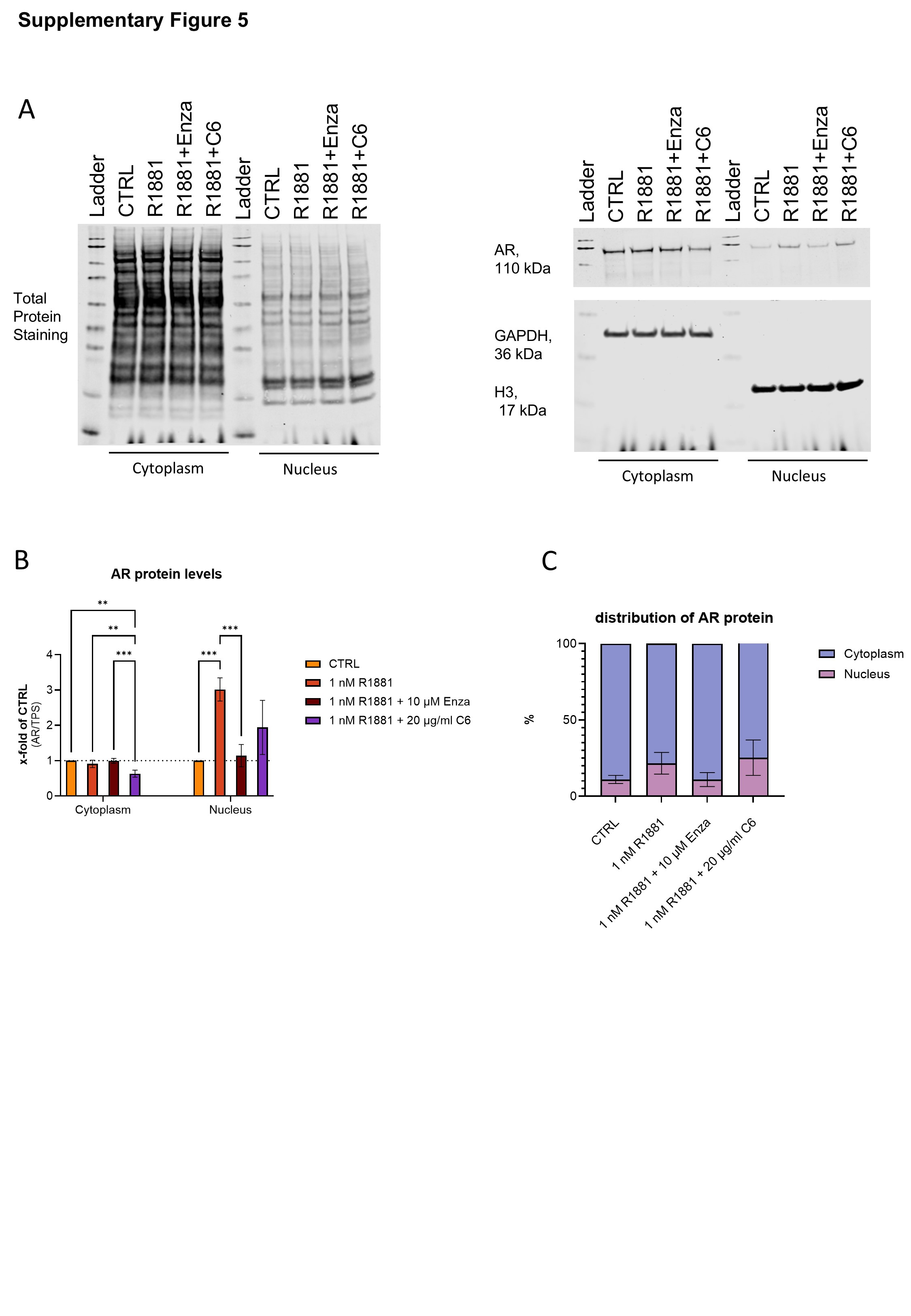

Supplement: Supplementary file 5 — Supplementary Fig. 5: (A) Representative western blot after cytoplasmic and nuclear extraction to investigate the localisation of the AR after treatment with DMSO (CTRL), 1 nM R1881, 1 nM R1881 + 10 µM Enzalutamide, and 1 nM R1881 + 20 µg/ml Cycloheximide. (B) Densitometry of AR protein levels relative to TPS in the cytoplasm and nucleus of LNCaP. Relative expression levels after treatment were shown as mean ± SD of six independent experiments. All differences highlighted by asterisks were statistically significant (**: p ≤ 0.01; ***: p ≤ 0.001). (C) Percentage distribution of AR protein in the cytoplasmic and nuclear fractions. [file 13062_2024_550_MOESM5_ESM.jpg]

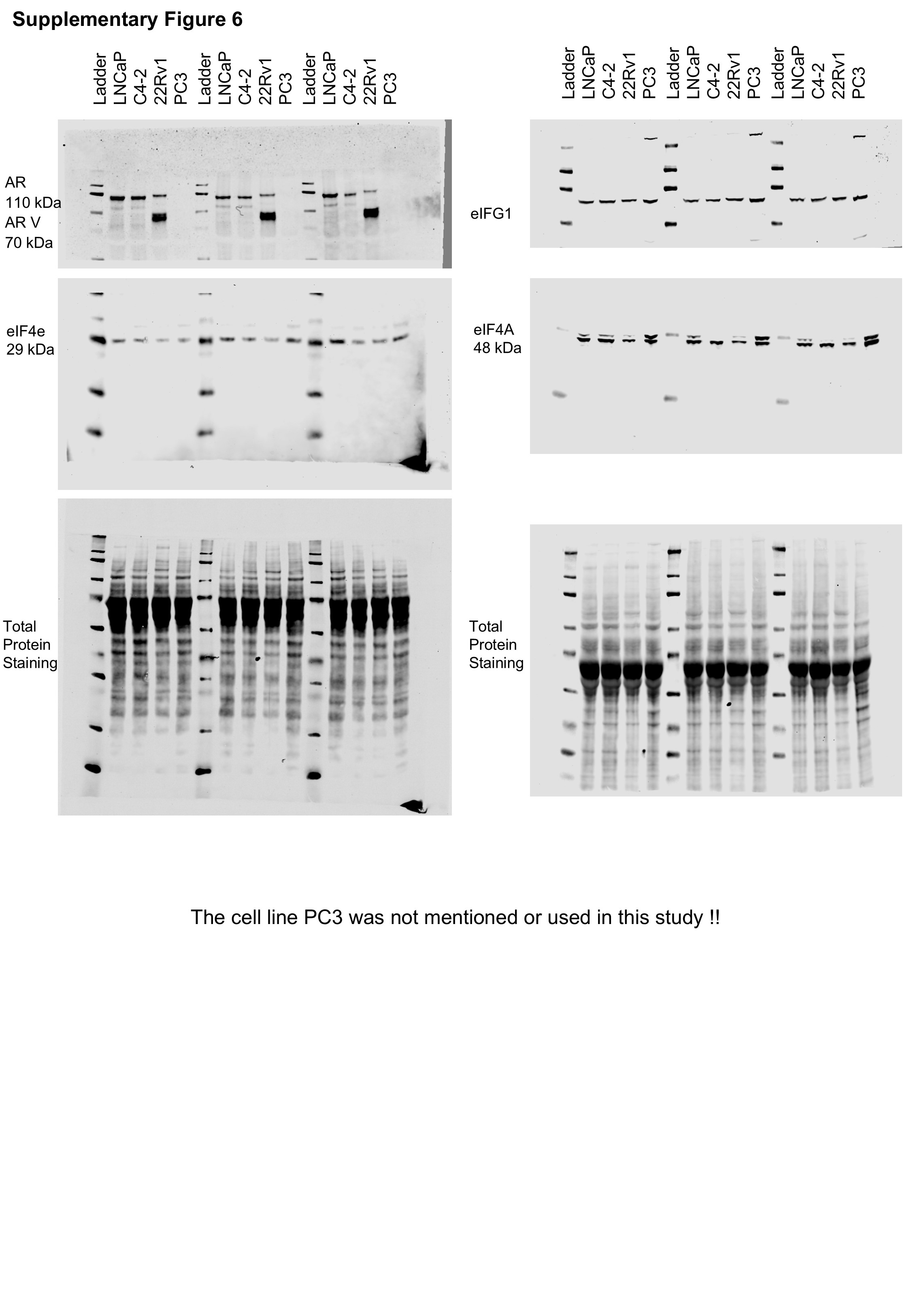

Supplement: Supplementary file 6 — Supplementary Fig. 6: (A + B) Uncropped Western blots for Fig. 4B. [file 13062_2024_550_MOESM6_ESM.jpg]

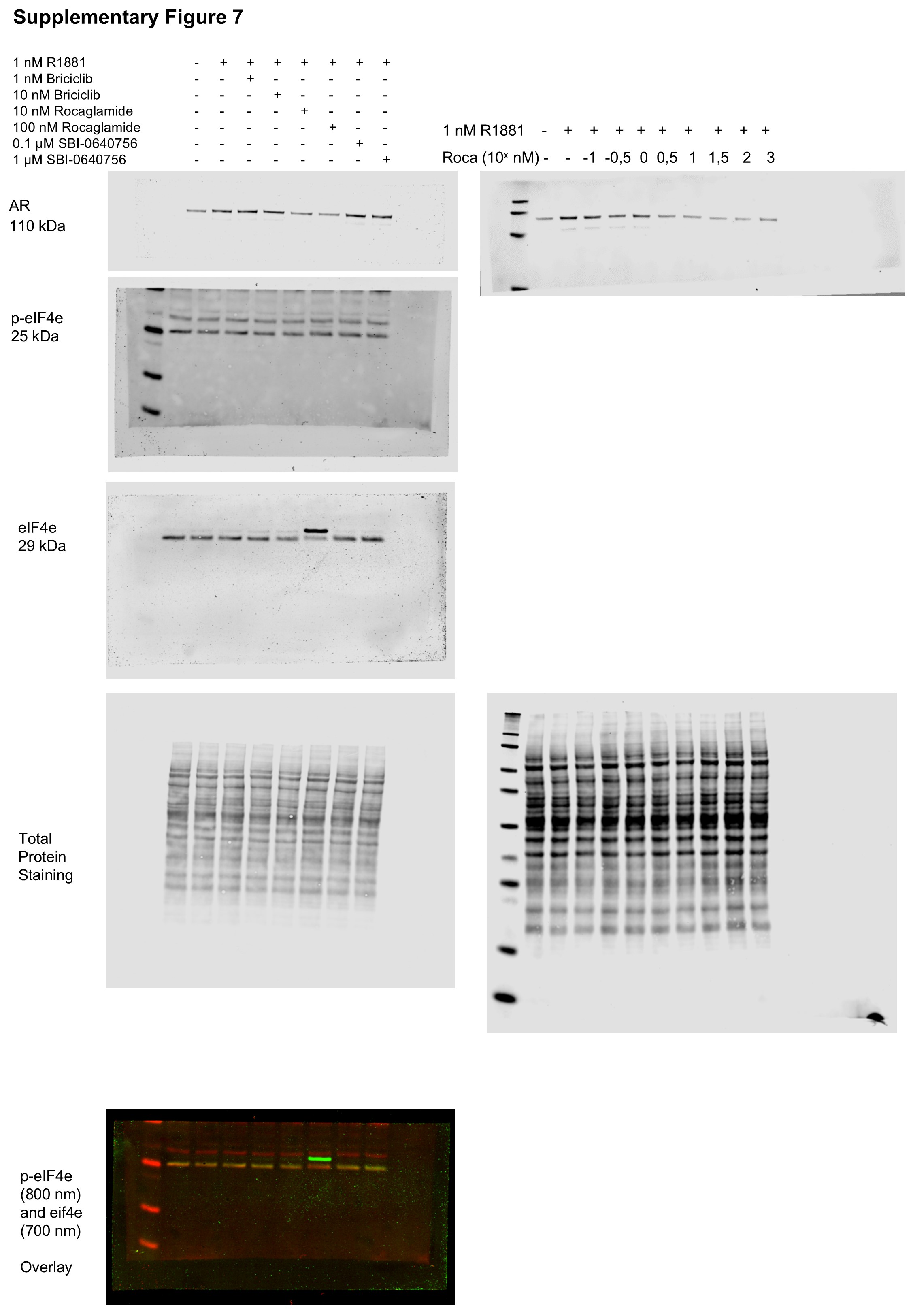

Supplement: Supplementary file 7 — Supplementary Fig. 7: (A) Uncropped Western blots for Fig. 5A. (B) Uncropped Western blots for Fig. 5E. [file 13062_2024_550_MOESM7_ESM.jpg]

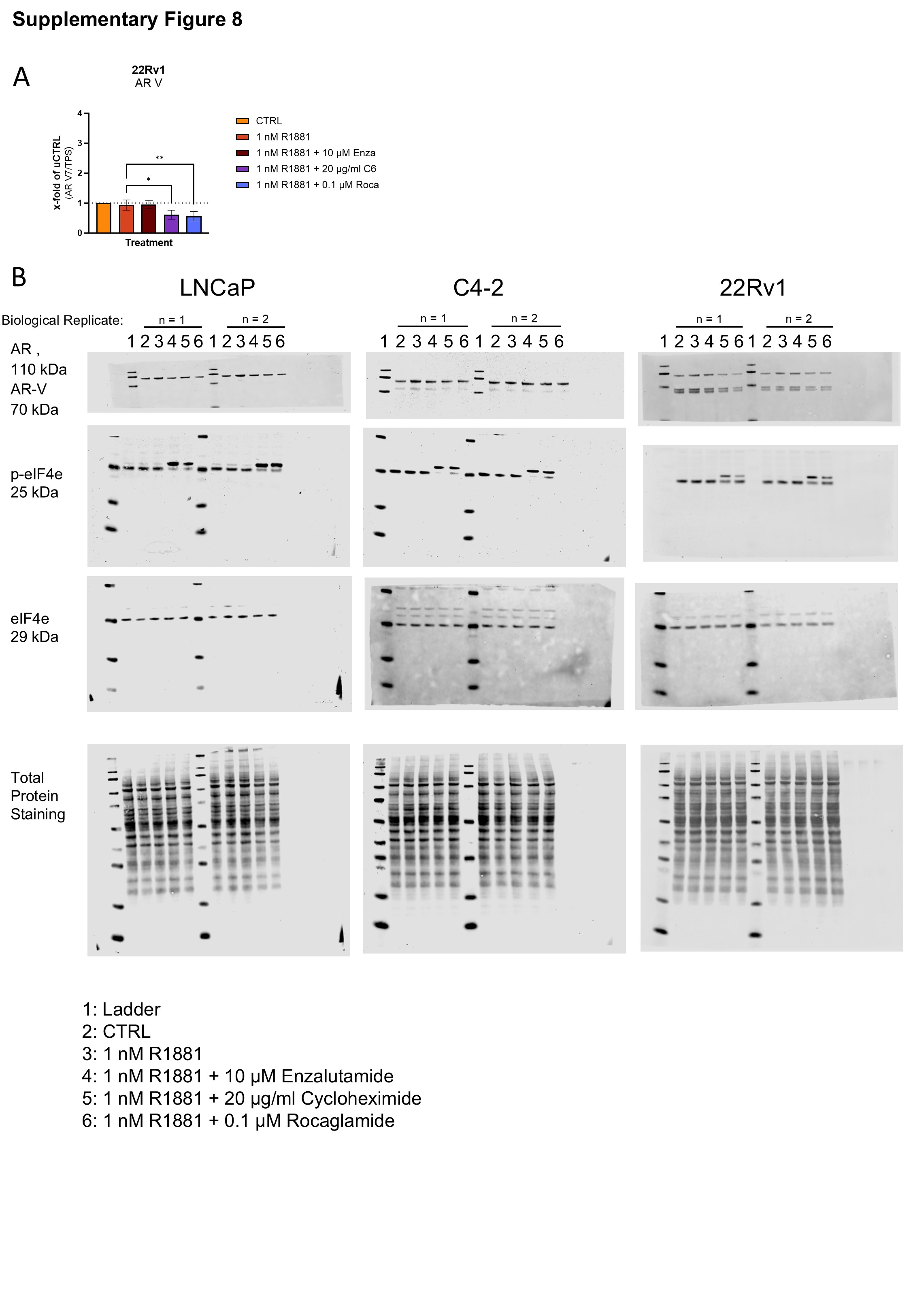

Supplement: Supplementary file 8 — Supplementary Fig. 8: (A) Densitometry of AR V protein levels relative to TPS in LNCaP, C4-2, and 22Rv1 cells. Relative expression levels after treatment were shown as mean ± SD of six independent experiments. All differences highlighted by asterisks were statistically significant (**: p ≤ 0.01; ***: p ≤ 0.001). (B) Uncropped Western blots for Fig. 6A. [file 13062_2024_550_MOESM8_ESM.jpg]
